# The Retromer Complex Is Required for Rhodopsin Recycling and Its Loss Leads to Photoreceptor Degeneration

**DOI:** 10.1371/journal.pbio.1001847

**Published:** 2014-04-29

**Authors:** Shiuan Wang, Kai Li Tan, Melina A. Agosto, Bo Xiong, Shinya Yamamoto, Hector Sandoval, Manish Jaiswal, Vafa Bayat, Ke Zhang, Wu-Lin Charng, Gabriela David, Lita Duraine, Kartik Venkatachalam, Theodore G. Wensel, Hugo J. Bellen

**Affiliations:** 1 Program in Developmental Biology, Baylor College of Medicine, Houston, Texas, United States of America; 2 Department of Biochemistry and Molecular Biology, Baylor College of Medicine, Houston, Texas, United States of America; 3 Department of Human and Molecular Genetics, Baylor College of Medicine, Houston, Texas, United States of America; 4 Jan and Dan Duncan Neurological Research Institute, Texas Children's Hospital, Houston, Texas, United States of America; 5 Howard Hughes Medical Institute, Baylor College of Medicine, Houston, Texas, United States of America; 6 Program in Structural and Computational Biology and Molecular Biophysics, Baylor College of Medicine, Houston, Texas, United States of America; 7 Department of Integrative Biology and Pharmacology, University of Texas School of Medicine, Houston, Texas, United States of America; 8 Department of Neuroscience, Baylor College of Medicine, Houston, Texas, United States of America; New York University, United States of America

## Abstract

Rhodopsin mistrafficking can cause photoreceptor (PR) degeneration. Upon light exposure, activated rhodopsin 1 (Rh1) in *Drosophila* PRs is internalized via endocytosis and degraded in lysosomes. Whether internalized Rh1 can be recycled is unknown. Here, we show that the retromer complex is expressed in PRs where it is required for recycling endocytosed Rh1 upon light stimulation. In the absence of subunits of the retromer, Rh1 is processed in the endolysosomal pathway, leading to a dramatic increase in late endosomes, lysosomes, and light-dependent PR degeneration. Reducing Rh1 endocytosis or Rh1 levels in retromer mutants alleviates PR degeneration. In addition, increasing retromer abundance suppresses degenerative phenotypes of mutations that affect the endolysosomal system. Finally, expressing human Vps26 suppresses PR degeneration in *Vps26* mutant PRs. We propose that the retromer plays a conserved role in recycling rhodopsins to maintain PR function and integrity.

## Abstract 2

Rhodopsin recycling via the retromer, rather than degradation through lysosomes, can alleviate light-induced photoreceptor degeneration in *Drosophila*.

## Author Summary

Upon light exposure, rhodopsins—light-sensing proteins in the eye—trigger visual transduction signaling to activate fly photoreceptor cells. After activation, rhodopsins can be internalized from the cell surface into endosomes and then degraded in lysosomes. This mechanism prevents constant activation of the visual transduction pathway, thereby maintaining the function and integrity of photoreceptor cells. It is not known, however, whether these internalized rhodopsins can be recycled. Here, we show that the retromer, an evolutionarily conserved protein complex, is required for the recycling of rhodopsins. We find that loss of key retromer subunits (Vps35 or Vps26) causes rhodopsin mislocalization in the photoreceptors and severe light-induced photoreceptor degeneration. Conversely, gain of retromer subunits can alleviate photoreceptor degeneration in some contexts. Human retromer components can stand in for depleted fruit fly retromer, suggesting that this complex plays a role in recycling light sensors in both vertebrate and invertebrate photoreceptors.

## Introduction

Rhodopsins are G protein-coupled receptors that function as light sensors in photoreceptors (PRs), and defective trafficking of rhodopsins often leads to PR degeneration in humans and flies [1]–[5]. Because vision is not required for animal survival, previous studies in *Drosophila* mostly focused on viable mutations that specifically impair PR function [1]. However, it is likely that numerous additional players encoded by essential genes have remained unidentified. We performed an eye-specific mosaic genetic screen [6] and found that loss of subunits of the retromer causes light-induced PR degeneration.

**Figure 1:**
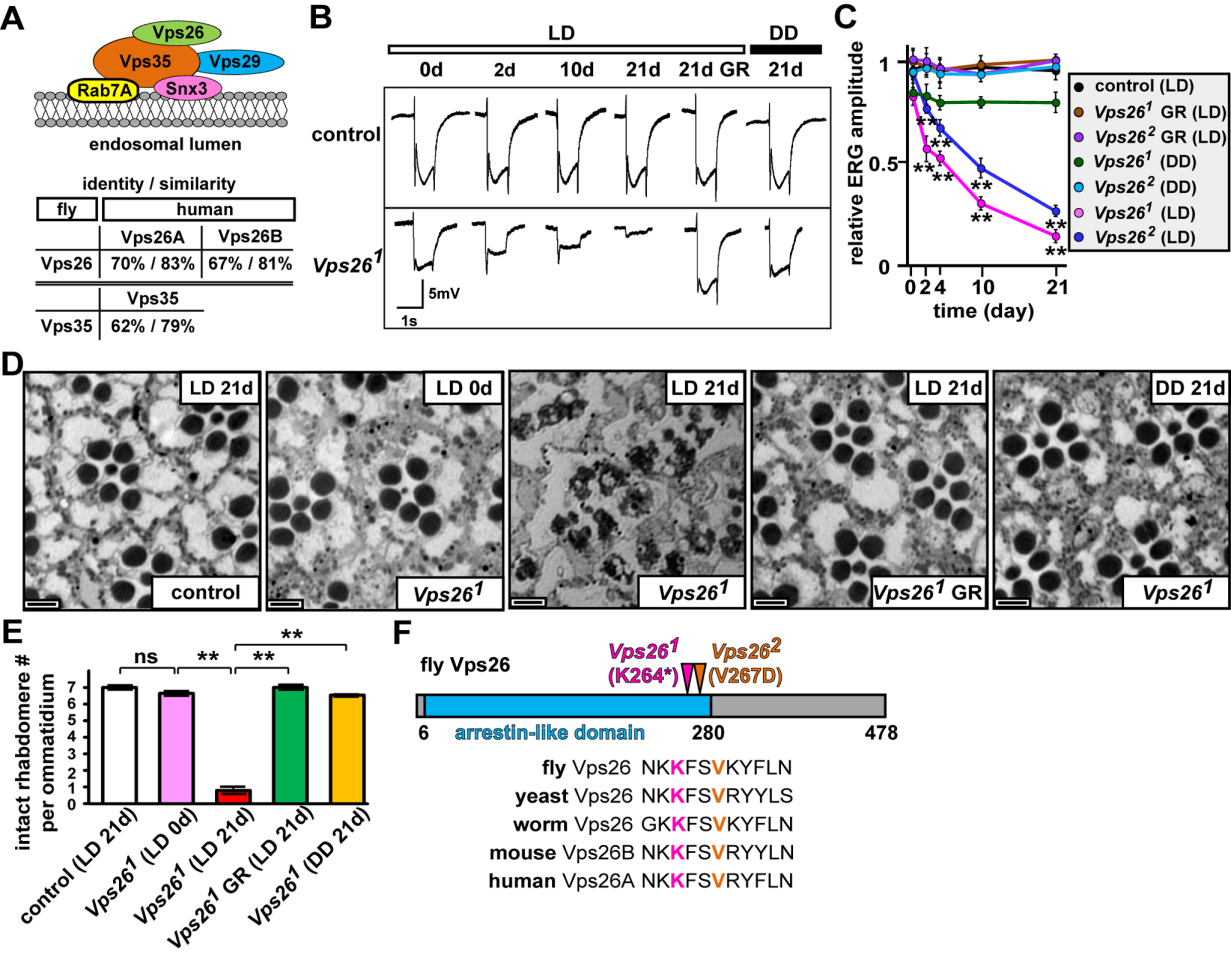
Loss of Vps26 in the eye causes PR degeneration. (A) Schematic showing the retromer complex associated with an endosomal membrane. Snx3 and Rab7A recruit the Vps35-29-26 trimer to endosomes. Vps35 is one of the protein partners of Vps26 in the retromer. The arrestin-like domain of Vps26 and the α-solenoid domain of Vps35 are aligned with the human homologs. (B) ERG traces of control (iso) and *Vps26^1^* mosaic eyes at day 0, kept in LD (light intensity = 1,800 lux) for 2, 10, and 21 d; *Vps26^1^* rescued by an EGFP-tagged genomic rescue construct (GR, *Vps26-gEGFP*) kept in LD for 21 d; and *Vps26^1^* kept in DD for 21 d. A UAS-RNAi against the *w* gene product is expressed in the eyes of the rescued animals (*Vps26^1^* GR or *Vps26^2^* GR) to remove red pigments. On- and off-transients disappear and the amplitude decreases upon 2 d in LD, but remain unchanged upon keeping the flies in DD for 3 wk. The loss of on- and off-transients and reduced depolarization can be fully rescued by the *Vps26-gEGFP* rescue construct. (C) Quantification of ERG amplitudes of flies kept in LD or DD. Ten ERG traces were recorded for each genotype at the indicated time points. ERG amplitudes are normalized to control PRs recorded at day 0. Light responses of *Vps26^1^* (or *Vps26^2^*) are compared between the mutants kept in LD and those in DD at indicated time points. Student's *t* test; error bars represent SEM; **p<0.01. (D) Bright field sections of control, *Vps26^1^*, and rescued *Vps26^1^* flies kept in LD for the indicated periods, or *Vps26^1^* kept in DD for 21 d. The UAS-*w* RNAi is expressed in the rescued animals. Newly eclosed *Vps26^1^* mutants are mostly normal but exhibit an occasional loss of a rhabdomere. The PRs of *Vps26^1^* are severely impaired after 21 d in LD but remain largely intact in DD. The *Vps26-gEGFP* rescue construct rescues the PR degeneration of *Vps26^1^* mutants. Scale bar, 2 µm. (E) Quantification of intact rhabdomere numbers in (D). Thirty ommatidia from three animals were examined for each genotype. Student's *t* test; error bars are SEM; ** *p*<0.01; ns, no significance. (F) Molecular lesions in *Vps26*. Mutations in both alleles alter highly conserved amino acid residues in an arrestin-like domain.

The retromer, a hetero-multimeric protein complex, retrieves specific proteins from endosomes, thereby preventing the degradation of these proteins in lysosomes [7]–[9]. The retromer is composed of Vps26, Vps29, Vps35, and certain sorting nexins (Snx) (Figure 1A, [7]–[9]). Most subunits are evolutionarily conserved (Figure 1A, [7]–[9]). Mutations in some subunits (Vps35 or Snx3) of the retromer have been shown to decrease the abundance of Wntless (Wls) and impair the secretion of Wingless (Wg), a ligand of the Wnt signaling pathway [10]–[14]. Wls is a transmembrane protein that binds to Wg and is required for Wg secretion [15],[16]. Impaired retromer function leads to excessive degradation of Wls in lysosomes, severely reducing Wg secretion and signaling [10]–[14]. The retromer has also been shown to maintain the levels of Crumbs, a transmembrane protein required for maintaining the apicobasal polarity in some tissues [17],[18]. More recently, mutations in human *VPS35* have been shown to cause a dominant inherited form of Parkinson's disease (PD) [19],[20]. However, the retromer has not been implicated in neurons of the visual system in flies or vertebrates.

The *Drosophila* compound eye comprises ∼800 hexagonal units named ommatidia [1],[2],[21],[22]. Each ommatidium contains eight PRs (R1–R8) that express rhodopsin proteins [1],[2],[21]–[23]. Rhodopsin 1 (Rh1) is the major rhodopsin that is primarily expressed in R1–R6 [1],[2],[21],[22]. It is synthesized and folded in the endoplasmic reticulum (ER) and transported to rhabdomeres, the stacked membranous structures in PRs, via the secretory pathway [1],[2],[21]. The proper transport of Rh1 from ER to rhabdomeres requires molecular chaperones [24]–[30] and Rab GTPases [24]–[33]. Binding of opsins to chromophores [34]–[40] as well as protein glycosylation and deglycosylation [41]–[44] are essential for Rh1 folding and maturation. Mutations in genes involved in Rh1 synthesis, folding, or transport can result in defective PR development or PR degeneration [24],[25],[32],[41]–[43],[45]–[51].

Phototransduction in the PRs relies on the activation of Rh1 by photons (Figure S1A, [52]). Active Rh1 (metarhodopsin, M*) activates phospholipase C (PLC) [53], which hydrolyzes phosphatidylinositol 4,5-bisphosphate (PIP_2_) to produce diacylglycerol (DAG) [54]. DAG or its metabolites can activate Transient Receptor Potential (TRP) and TRP-like cation channels that lead to depolarization of the PRs [55]–[59]. Similar to fly PRs, the vertebrate intrinsically photosensitive retinal ganglion cells (ipRGCs) use melanopsin (a homolog of fly Rh1) as the light sensor and requires PLC and TRPC channels for activation [60]–[63]. ipRGCs project their axons to specific brain areas to control circadian rhythms [64]–[67] or pupillary light reflex [64],[68],[69].

Tight regulation of Rh1 activity upon light exposure is required to maintain the integrity of PR cells [1],[2]. M* can be converted into its inactive form upon exposure to orange light [52]. In addition, a significant portion of active Rh1 can be endocytosed and degraded in lysosomes [70]–[72]. Mutations that abolish Rh1 deactivation [72]–[74] or impair the endolysosomal system [71],[75],[76] can cause PR degeneration due to Rh1 accumulation. However, it is unknown whether Rh1 can be retrieved from the endolysosomal compartments and whether impaired Rh1 recycling leads to PR degeneration.

Here, we show that loss of the fly Vps26 or Vps35 causes early-onset PR degeneration. We show that retromer subunits are expressed in PRs in flies and melanopsin-expressing ipRGCs in the mouse retina. In fly mutant PRs, the numbers of late endosomes and lysosomes are significantly elevated. The PR degenerative phenotypes are dependent on exposure to light and the presence of Rh1. Our data indicate that the fly retromer recycles Rh1, preventing Rh1 retention in the PR cell bodies and shunting Rh1 from being degraded in lysosomes, thereby promoting Rh1 redelivery to rhabdomeres. In summary, the retromer recycles Rh1, prevents an overload of the endolysosomal pathway, and salvages a substantial fraction of Rh1 from degradation in flies. It may also play a similar role in ipRGCs in the retina of vertebrates.

## Results

### *Vps26* Mutant PRs Display Light-Dependent PR Degeneration

To identify mutations that cause PR degeneration, we generated large mutant clones of essential genes in the eyes (Figure S1B) and screened for mutants that exhibit an age-dependent decrease in amplitude of electroretinogram (ERG) recordings [6],[77],[78]. Mutations in a complementation group *XE52*, which we later mapped to *Vps26* (Figure S2A), result in a progressive loss of ERG amplitudes compared to the control (Figure 1B–C and Figure S1C). Reduced amplitudes in ERG recordings may result from aberrant PR development, a defective phototransduction pathway, or PR degeneration [1]. To address if the ERG defects are due to aberrant PR development, we examined the light response of dark-reared control (*y w FRT19A^iso^*, the isogenized *y w FRT19A* flies, or iso; see Materials and Methods) and two *Vps26* mutant alleles. Upon rearing the flies in the dark, the *Vps26^1^* and *Vps26^2^* mutants exhibit nearly normal ERG responses and the ERG amplitude and on- and off-transients in *Vps26^1^* mutant PRs are slightly reduced when compared to control (Figure 1B–C and Figure S1C). In addition, imaging of newly eclosed flies (see definition in Materials and Methods) revealed that the overall morphology of ommatidia is similar to control, although loss of a rhabdomere is observed at low frequency in the *Vps26^1^* mosaic clones (Figure 1D–E and Figure S1D–E). These data indicate that mutations in *Vps26* do not significantly affect PR development, allowing nearly normal ERGs in young flies.

To assess the impact of the *Vps26* mutations in aged flies, we raised the animals either in a 12-h light/dark cycle (LD, L = 1,800 lux) or in constant darkness (DD) and recorded the ERGs at 0, 2, 4, 10, and 21 d. In LD, both alleles show a loss of on- and off-transients, indicating a loss of synaptic transmission. They also exhibit a reduced depolarization by day 2, and a progressive loss of the ERG amplitude with age (Figure 1B–C and Figure S1C). At day 21, the light-induced PR depolarization in *Vps26^1^* and *Vps26^2^* mutant PRs is almost completely absent (Figure 1B–C and Figure S1C). Strikingly, 3-wk-old DD-reared *Vps26^1^* and *Vps26^2^* mutants exhibit nearly normal ERG responses, similar to newly eclosed flies (Figure 1B–C and Figure S1C). The PR morphology of *Vps26^1^* and *Vps26^2^* mutants is severely affected in flies kept in LD for 21 d (Figure 1D–E and Figure S1D–E). In addition, we constructed an EGFP-tagged genomic rescue construct (*Vps26-gEGFP*; see below and Figure S2A) that fully rescues the lethality (Figure S2B), ERG, and morphological defects of both *Vps26^1^* and *Vps26^2^* alleles (*Vps26^1^* GR in Figure 1B–E; *Vps26^2^* GR in Figure 1C and S1C–E). However, PR morphology is nearly wild-type when the flies are raised in DD (Figure 1D–E and Figure S1D–E). In summary, PR degeneration in the *Vps26* mutant clones is light-dependent.

### Mutations in *XE52* Map to *Vps26*

To identify the mutations in *XE52* complementation group consisting of two alleles, we performed duplication, deficiency, and P[acman] rescue mapping (Yamamoto et al., in preparation), narrowing the locus to a 21 kb genomic region (Figure S2A). The *XE52* alleles fail to complement the lethality of *P{EP}Vps26^G2008^* (Figures S2A–B, [79]), a transposable element inserted in the 5′-UTR of the *Vps26* gene. Sanger sequencing revealed mutations in conserved amino acid residues in both alleles: a nonsense mutation (K264*) and a missense mutation (V267D) in *Vps26^1^* and *Vps26^2^*, respectively (Figure 1F). To test if *Vps26* is responsible for both the lethality and PR degenerative phenotypes in *XE52* alleles, we generated the *Vps26-gEGFP* genomic rescue construct mentioned above (Figure S2A) as well as a *UAS-Vps26* full-length cDNA construct [80]. Both the genomic rescue and ubiquitously expressed cDNA (*tub-Gal4>UAS-Vps26*) rescue all phenotypes (Figure 1B–E, Figure S1C–E, Figure S2B, and unpublished data), confirming that the phenotypes are caused by loss of *Vps26*.

Vps26 is a subunit of the retromer (Figure 1A, [81],[82]). Because loss of retromer function in *Vps35* and *Snx3* mutants has been previously documented to lead to defects in Wg signaling [10]–[14], we tested whether the *Vps26* mutants isolated in our screen also exhibit this phenotype. We generated mutant clones of *Vps26^1^* in wing discs of third instar larvae and observed a failure to secrete Wg in the wing margin cells. Furthermore, we observed concomitant loss of Senseless (Sens) expression, a downstream effector of the Wingless pathway (Figure S2C, [83]). These data confirm the loss of retromer function in the *Vps26* mutants.

### Vps26 Is Primarily Localized to Early and Late Endosomes

The subunits of the retromer complex have been shown to primarily associate with endosomes in vertebrates [7]–[9]. However, the *in vivo* subcellular localization of the retromer in *Drosophila* has not been documented. We therefore raised a polyclonal antibody against the full-length fly Vps26 protein (Figure S2D–E) and costained with antibodies against Avalanche (Avl, fly homolog of Syntaxin 7, an early endosomal marker, [84]), Rab5 (an early endosomal marker, [85]), Rab7 (a late endosomal marker, [76]), Protein Disulfide Isomerase (PDI, an ER marker, [86]), GM130 (a *cis*-Golgi marker, [87]), Peanut Agglutinin (PNA, a marker for the *trans*-Golgi network (TGN), [88]), or Rab11 (a TGN or post-Golgi secretory vesicle marker, [31]). Vps26 shows substantially greater colocalization with Avl, Rab5, and Rab7 than the ER, Golgi, and secretory vesicle markers (Figure S2F–G). This distribution is seen in both wing discs and adult PRs (Figure S2F–G and unpublished data), indicating that Vps26 associates preferentially with early and late endosomes in different tissues *in vivo*.

### Loss of *Vps35* Causes PR Degeneration

**Figure 2:**
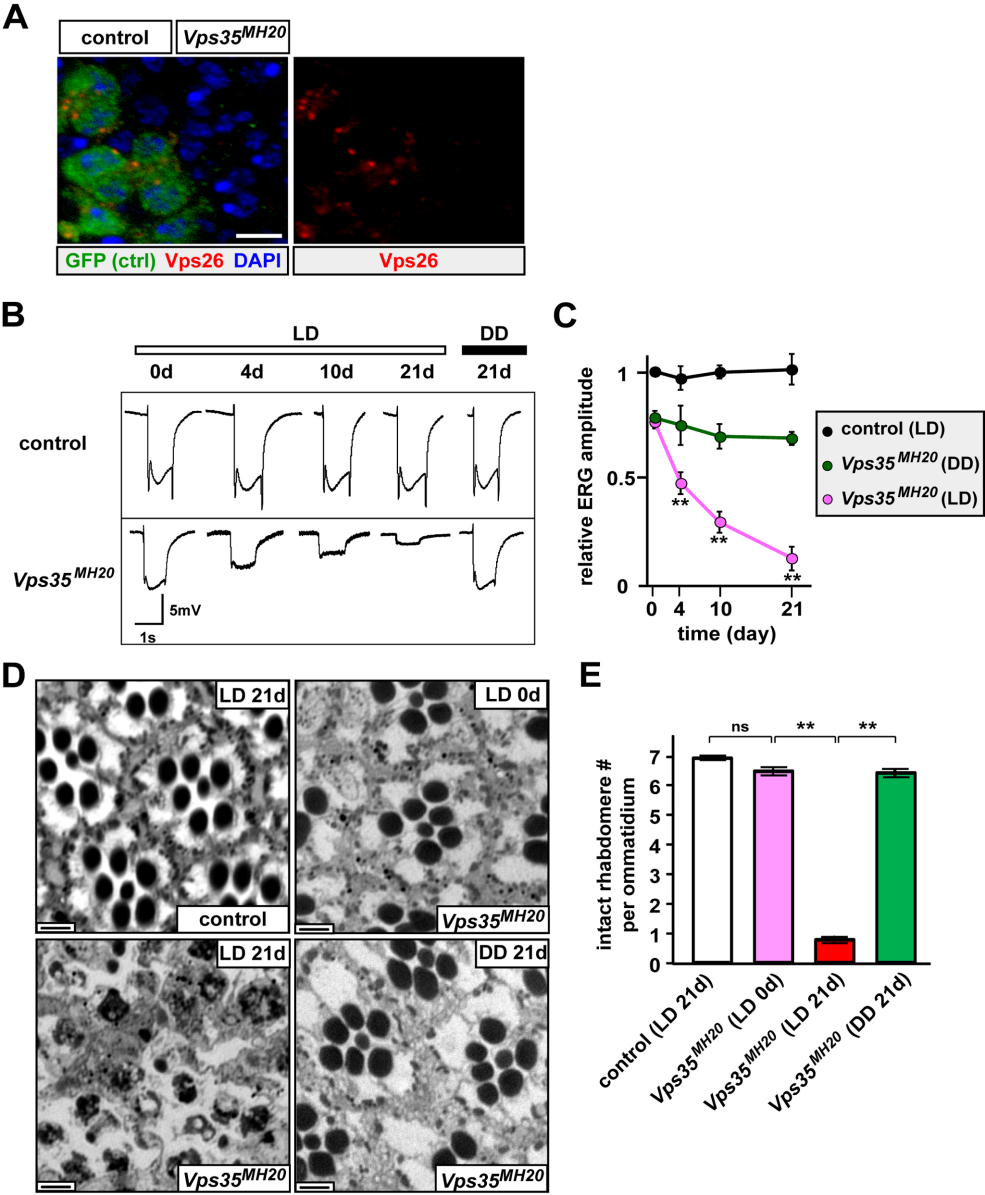
Loss of Vps35 leads to PR degeneration. (A) Vps26 immunostaining in *Vps35^MH20^* mosaic clones generated by *ey-FLP*. Differentiating cells posterior to the morphogenetic furrow in eye imaginal discs of the third instar larvae are examined. Control cells are marked by GFP, whereas *Vps35^MH20^* mutant cells lack GFP expression. Note the loss of Vps26 in *Vps35^MH20^* mutant cells, suggesting that the presence of the Vps26 protein is dependent on the presence of Vps35. Scale bar, 10 µm. (B) ERG traces of control (*FRT42D*) and *Vps35^MH20^* kept in LD for 0, 4, 10, or 21 d, or in DD for 21 d. *Vps35^MH20^* PRs lose on- and off-transients and exhibit a progressive loss of amplitude in LD but remain only mildly impaired in DD. (C) Quantification of ERG amplitude of the flies measured in (B). ERGs of 10 flies were quantified for each genotype at the indicated time points. ERG amplitudes are normalized to control PRs recorded at day 0 in LD. Depolarization is compared between *Vps35^MH20^* mutant PRs that are kept in LD and those in DD at the indicated time points. Student's *t* test; error bars represent SEM; ** *p*<0.01. (D) Bright field images of control and *Vps35^MH20^* kept in LD for the indicated periods, or *Vps35^MH20^* kept in DD for 21 d. Dark-reared *Vps35^MH20^* mutant at day 0 exhibits normal PR morphology. The PRs of *Vps35^MH20^* are severely impaired after 21 d in LD but remain largely intact in DD. Scale bar, 2 µm. (E) Quantification of intact rhabdomere numbers in (D). Thirty ommatidia from three animals were examined for each genotype. Student's *t* test; error bars represent SEM; ** *p*<0.01; ns, no significance.

Vps35 acts as a scaffolding protein and physically interacts with Vps26 in the retromer (Figure 1A, [81],[82],[89]). RNAi knockdown of *vps35* has been shown to reduce Vps26 protein levels in HEK293 cells [13]. Consistent with work in cultured cells, we find that mutant clones of the *Vps35* null allele (*Vps35^MH20^*, [13]) in eye discs cause a nearly complete loss of Vps26 (Figure 2A), showing that the presence of Vps35 is required for Vps26 *in vivo*. Next, we performed ERG recordings and bright field imaging on large mutant clones of *Vps35^MH20^* in flies aged for 0, 4, 10, and 21 d and examined function and morphology of *Vps35^MH20^* mutant PRs. As shown in Figure 2B–C, the ERG phenotypes of *Vps35^MH20^* are very similar to those of *Vps26* shown in Figure 1. In addition, mutations in the two genes exhibit very similar morphological defects in aged PRs (compare Figure 2D–E with Figure 1D–E and Figure S1D–E). These results indicate that the retromer is required to maintain PR function and morphology. Loss of the retromer therefore leads to light-dependent PR degeneration.

Sorting nexins can be involved in retromer-mediated protein trafficking [7]–[9],[90],[91]. To test whether these proteins are involved in the maintenance of PRs, we performed ERGs on *Snx1^d1^* and *Snx3^d1^* null alleles (Figure S3, [10]). Upon keeping the flies for 10 d in LD, we did not observe a difference in ERG amplitudes of *Snx1^d1^* or *Snx3^d1^* mutants (Figure S3A–B), although we observed a partial loss of the on-transient in *Snx3^d1^* (Figure S3A,C). The data indicate that these sorting nexins are not part of the complex or play a minor role. In addition, we tested whether the WASH (Wiskott–Aldrich Syndrome protein and SCAR Homolog) complex, a protein complex that has been shown to be required for the transport of several retromer cargos in vertebrate cells and the fly tracheal tubes [8],[90],[91], is involved in PR degeneration. As shown in Figure S4, RNAi-mediated loss of a subunit of the WASH complex, Washout (the single WASH ortholog in *Drosophila*, [92]), does not affect PR maintenance and function. Hence, the retromer can have different compositions in different cells.

### Loss of Retromer Subunits Leads to an Expansion of the Endolysosomal Compartments in PRs

**Figure 3:**
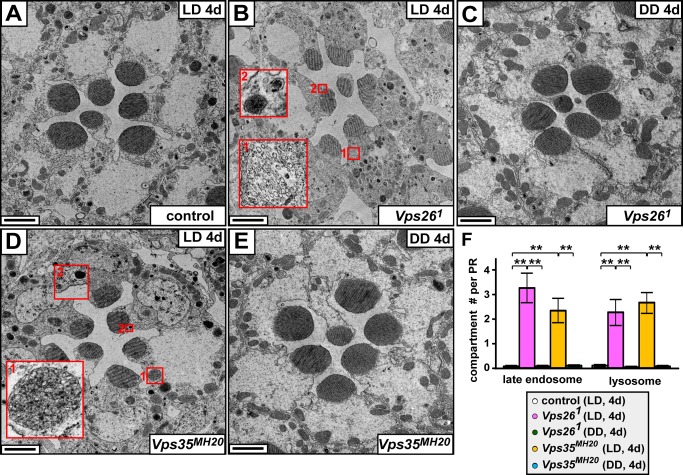
Late endosomes and lysosomes are expanded in *Vps26* and *Vps35* mutant PRs. (A) TEM of the PRs of a control (iso) fly kept in LD for 4 d show normal morphology with intact rhabdomeres and cell bodies. Scale bar, 1.5 µm. (B) Upon 4 d in LD, *Vps26^1^* mutant PRs exhibit highly aberrant morphological features: note the expansion of late endosomes (multivesicular structures, inset 1) and lysosomes (condensed structures, inset 2). In addition, electron dense particles are increased, and the morphology of some rhabdomeres is defective compared to control in (A). Scale bar, 1.5 µm. (C) Upon 4 d in DD, *Vps26^1^* mutant PRs exhibit similar morphology as controls. Scale bar, 1.5 µm. (D) Upon 4 d in LD, *Vps35^MH20^* mutant PRs show increased late endosomes (inset 1) and lysosomes (inset 2) similar to *Vps26^1^* mutants. Scale bar, 1.5 µm. (E) Upon 4 d in DD, the morphology of *Vps35^MH20^* mutant PRs remains similar to that of the control. Scale bar, 1.5 µm. (F) Quantification of the number of late endosomes and lysosomes in PRs at day 4 in LD or DD. Ten PRs were quantified for each genotype. Student's *t* test; error bars represent SEM; ** *p*<0.01.

The retromer retrieves membrane protein cargos from the endolysosomal pathway [7]–[9]. To assess whether endosomes and lysosomes are affected in the absence of the retromer, we performed transmission electron microscopy (TEM) and examined the ultrastructure of *Vps26* and *Vps35* mutant PRs. *Vps26^1^* and *Vps35^MH20^* flies raised in LD for 4 d show obvious ultrastructural defects (Figure 3B,D) in PRs when compared to control (Figure 3A). These defects include a dramatic increase in late endosomes (inset 1 in Figure 3B,D, Figure 3F, and yellow arrowheads in Figure S5B,D; [93]) and lysosomes (inset 2 in Figure 3B,D, Figure 3F, and arrows in Figure S5B–C,E; [94]). However, PRs of the dark-reared *Vps26^1^* or *Vps35^MH20^* flies (Figure 3C,E–F and Figure S6B–E) do not show an increased number of late endosomes or lysosomes when compared to control (Figure 3A,F, Figure S5A, and Figure S6A). These data show that the endolysosomal pathway is severely affected in retromer mutants upon light exposure.

### Internalized Rh1 Fails to Be Recycled from the Endolysosomal Pathway in *Vps26* Mutant PRs

To address whether the trafficking of endocytosed Rh1 is affected in the retromer mutants, we assessed Rh1 distribution in PRs that were exposed to light. Two protocols have been used for Rh1 immunostaining in fly PRs: whole mount preparations [76] and eye sections [41]. Previously, we showed that the whole mount protocol is more sensitive than staining of sections for Rh1 present in the cell body, while sections are better than whole mount staining for assessing Rh1 in rhabdomeres [6]. Here, we slightly modified the whole mount protocol to increase Rh1 signals in the PR cell body by treating the tissues with higher concentrations of Triton X-100. As shown in Figure S7, Rh1 signals are obviously enhanced upon increasing Triton X-100 concentration (top panels) compared to low Triton X-100 treatment (bottom panels). Here, we use the high Triton X-100 protocol for our immunostaining assays.

**Figure 4:**
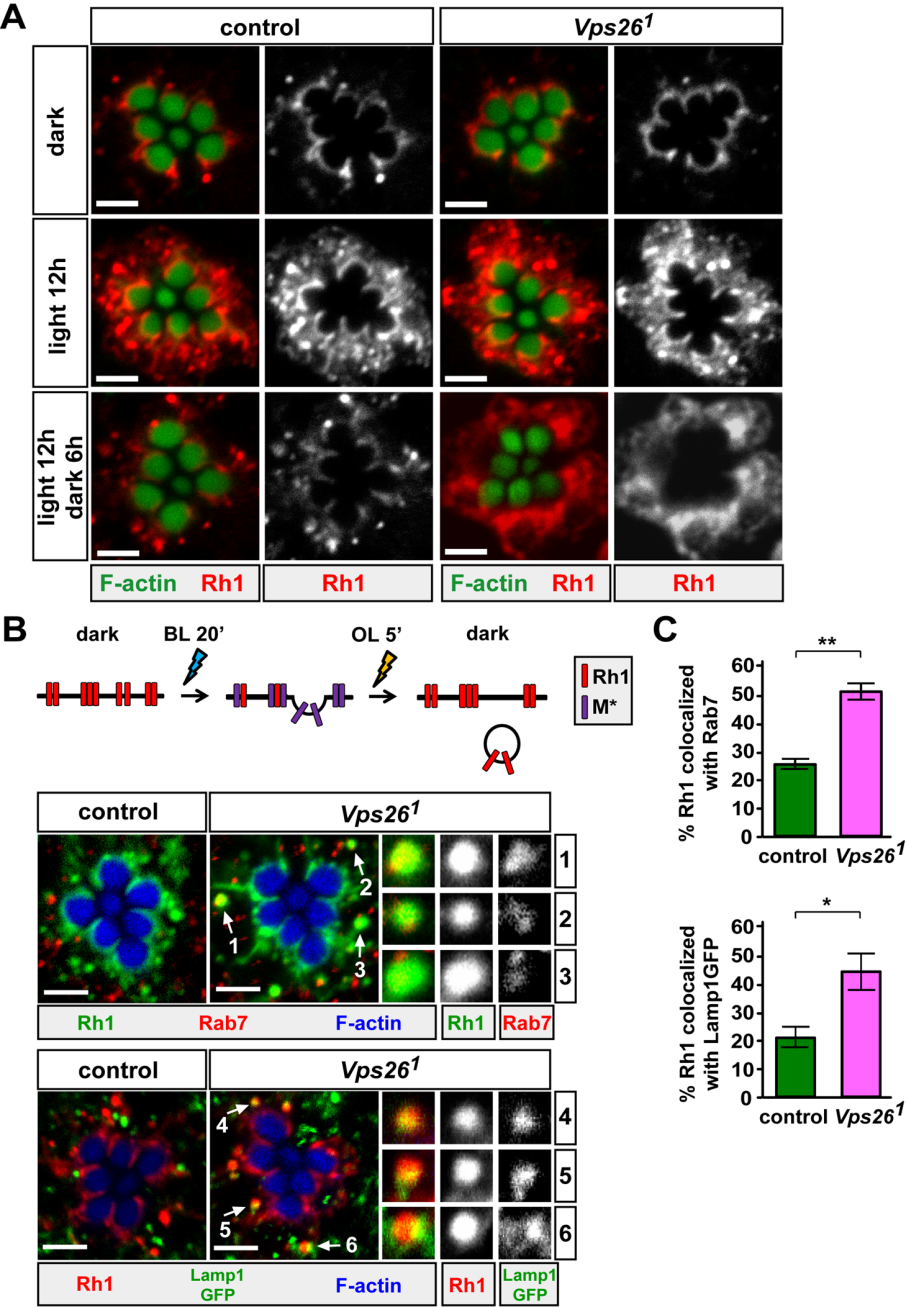
Internalized Rh1 accumulates in late endosomes in *Vps26* mutants. (A) Rh1 is aberrantly distributed in *Vps26^1^* mutants. Dark-reared control (iso) or *Vps26^1^* mutants were kept in the dark, exposed to whole-spectrum light for 12 h, or exposed to light for 12 h and recovered in the dark for 6 h. Both dark-reared control and *Vps26^1^* mutant PRs show a normal Rh1 distribution, which is characterized by the crescent-shaped Rh1 staining pattern surrounding rhabdomeres (marked by F-actin) (top panels). The localization of Rh1 in the shape of a crescent around the rhabdomeres is due to the dense packing of membranes in rhabdomeres. Upon a 12-h light exposure, both genotypes show increased Rh1 protein levels in PR cell body (middle panels) as some Rh1 is endocytosed. However, upon a 6-h recovery in the dark, the Rh1 signal in the cell body is higher in *Vps26^1^* than that in wild-type PRs (bottom panels). Scale bar, 2 µm. (B) Endocytosed Rh1 preferentially colocalizes with Rab7, a late endosomal marker, or Lamp1::GFP, a lysosomal marker. Schematic showing the Rh1 pulse-chase assay. Rh1 internalization is induced by a 20-min blue light (BL) pulse followed by a 5-min orange light (OL) exposure to inactivate Rh1. Subsequently, the flies are kept in the dark for 4 h to allow Rh1 delivery to endosomal compartments. An *w* RNAi is ectopically expressed in the eyes of the Lamp1::GFP-expressing control or *Vps26^1^* flies. Fly PRs are stained with phalloidin (to mark F-actin), Rh1, Rab7, or GFP. Rh1 is enriched in Rab7 (arrows 1–3) or Lamp1::GFP (arrows 4–6) punctae in *Vps26^1^* PRs. (C) Quantification of Rh1 punctae colocalized with Rab7 or Lamp1::GFP in control and *Vps26^1^* mutant PRs. Thirty ommatidia from five animals for each genotype were quantified. Student's *t* test; error bars represent SEM; * *p*<0.05; ** *p*<0.01.

**Figure 5:**
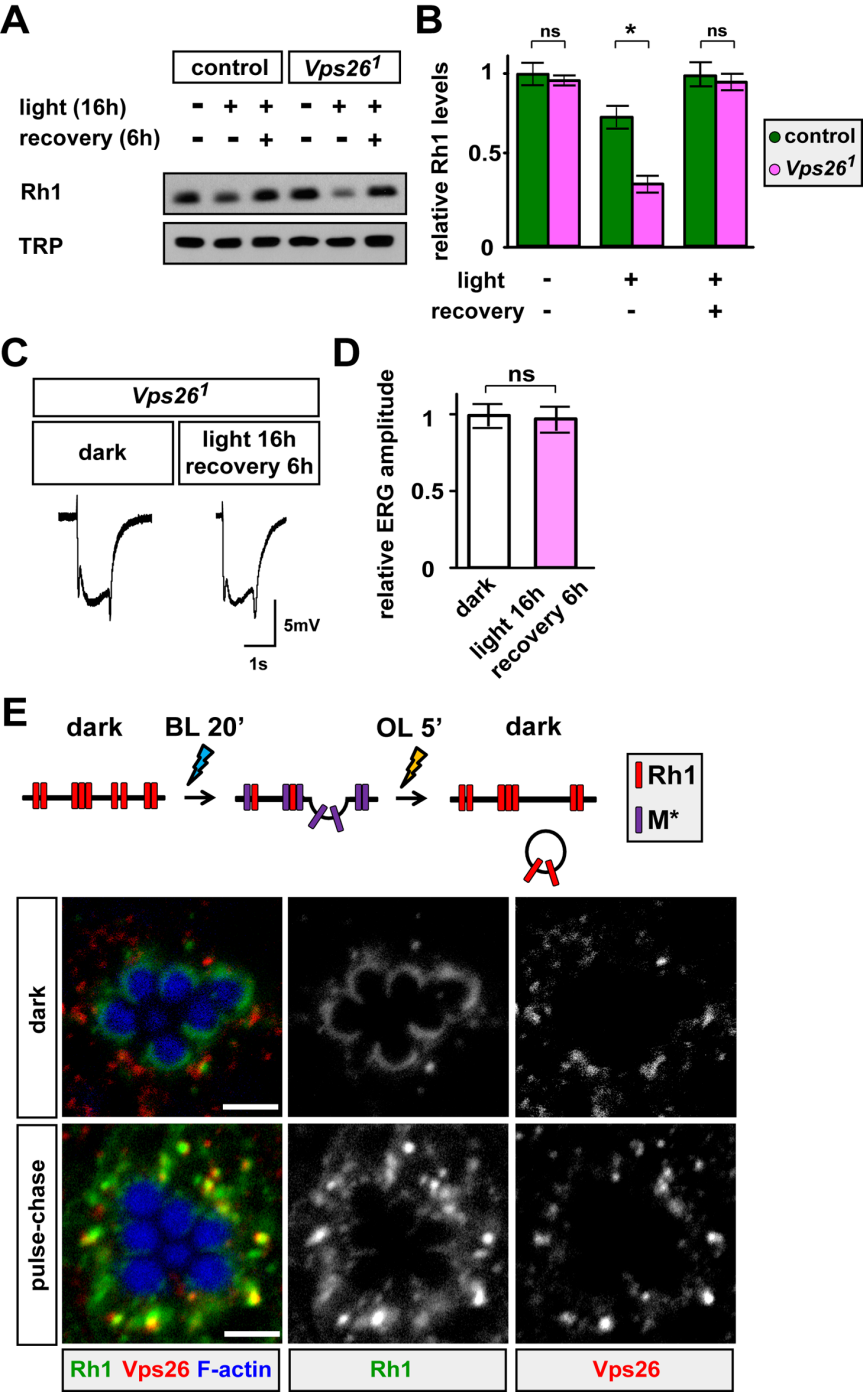
Rh1 levels are decreased in *Vps26* mutant PRs after internalization. (A) Western blots for Rh1 and TRP on control (iso) and *Vps26^1^* mutant PRs. Flies are kept in the dark, exposed to white light for 16 h to induce endocytosis and lysosomal degradation of Rh1, or exposed to 16-h white light and allowed to recover in the dark for 6 h. The dark-reared control and *Vps26^1^* mutants show similar Rh1 levels. Upon 16-h exposure to white light, Rh1 levels in *Vps26^1^* decrease more than those in control. After recovery in the dark, both control and *Vps26^1^* mutants exhibit comparable Rh1 levels. We loaded 0.4 fly heads in each lane. (B) Quantification of the levels of Rh1 normalized to TRP. Three independent experiments were performed. Student's *t* test; error bars represent SEM; * *p*<0.01; ns, no significance. (C) ERG traces of *Vps26^1^* mosaic flies kept in the dark, or exposed to light for 16 h and then recovered in the dark for 6 h. ERG responses of *Vps26^1^* mutant PRs are not impaired when exposed to these illumination paradigms. (D) Quantification of ERG amplitude in (C). ERGs of 10 flies were quantified for each condition. ERG amplitudes are normalized to dark-reared *Vps26^1^* mutant PRs. Error bars represent SEM; ns, no significance. (E) Rh1 colocalizes with Vps26 in control PRs. Rh1 internalization is induced by the pulse-chase protocol used in Figure 4B. Upon a 2-h recovery in the dark, control PRs are stained with Rh1 and Vps26 antibodies. Multiple Rh1 punctae colocalize with Vps26.

As previously reported [6],[76], immunostaining of Rh1 in dark-reared wild-type control PRs reveals a typical crescent-shaped staining pattern at the base of the rhabdomeres, due to the densely stacked rhabdomeric membranes that do not permit antibody penetration in whole mount preparations (Figure 4A, top panels). Similar to this control, we observed that the distribution (Figure 4A, top panels) and levels (Figure 5A–B) of Rh1 are not affected in *Vps26^1^* mutants prior to light exposure. These data are in agreement with the observation that dark-reared *Vps26^1^* mutants exhibit nearly normal ERGs and PR morphology (Figure 1B–E). Upon a 12-h light exposure, Rh1 is internalized in both control and *Vps26^1^* mutant PRs, as shown by the dramatic increase in Rh1 levels in the PR cell bodies (Figure 4A, middle panels). This increase in Rh1 is not due to the changes in levels of Rh1 (unpublished data), but rather because Rh1 becomes increasingly accessible to the antibodies upon endocytosis [6]. In addition, this light exposure is not pathological as control flies exhibit normal ERGs and PR morphology (Figure S8). However, upon a 6-h recovery in the dark, the Rh1 signal in the cell body is restored to the original levels in control but not in *Vps26^1^* PRs (Figure 4A, bottom panels). Similarly, although less obvious, we also observed Rh1 accumulation in *Vps26^1^* mutant PRs upon light exposure in the sectioned eye samples (Figure S9). Hence, the Rh1 subcellular localization is altered in *Vps26^1^* mutants.

To assess where in the cell body Rh1 accumulates in *Vps26^1^* mutants, we transiently exposed the flies to light and monitored the subcellular localization of Rh1 using markers. We briefly exposed the flies to blue light for 20 min followed by 5-min exposure to orange light (Figure 4B) to acutely induce endocytosis of a small amount of Rh1. This light condition is not pathological as the ERG responses and morphology of control PRs are normal (Figure S8). Upon recovery in the dark for 4 h, Rh1 can be detected in very bright punctae that often colocalize with Rab7 or Lamp1::GFP, a marker for lysosomes [95], in *Vps26^1^* PRs (Figure 4B–C), implicating that Rh1 is trapped in the endolysosomal compartments.

Rh1 retention in the endolysosomal pathway in *Vps26^1^* mutants upon an acute light exposure may be due to reduced Rh1 recycling and/or decreased lysosomal degradation. If Rh1 recycling is decreased, one would expect a reduction in Rh1 levels in *Vps26^1^* mutants compared to controls upon light exposure as an increased amount of internalized Rh1 will be delivered to lysosomes for degradation. On the other hand, if Rh1 degradation in lysosomes is impaired, one would predict that Rh1 abundance is elevated in *Vps26* mutant PRs when compared to controls exposed to light. To distinguish between these two possibilities, we exposed the dark-raised flies to white light (1,800 lux) for 16 h. Prior to this light exposure, the Rh1 levels are similar in control and *Vps26^1^* mutants (Figure 5A–B), indicating that Rh1 expression is not affected in *Vps26^1^* mutants. Upon a 16-h exposure to white light, Rh1 levels in *Vps26^1^* mutant PRs are significantly decreased when compared to control (Figure 5A–B). Reduced Rh1 levels in *Vps26^1^* mutants upon light exposure do not appear to be due to impaired Rh1 synthesis as the Rh1 levels are restored after 6-h recovery in the dark (Figure 5A–B). Moreover, the ERG responses of *Vps26^1^* mutants are not affected upon this light exposure (Figure 5C–D). The data indicate that, in the absence of Vps26, lysosomes are able to degrade internalized Rh1 to maintain PR function when briefly exposed to light. However, chronic light exposure (LD for several days) severely affects the endolysosomal system (Figure 3 and Figure S5) because accumulated Rh1 in endosomes and/or lysosomes persistently challenges the endolysosomal system [2].

To examine whether the retromer is recruited to Rh1 punctae upon endocytosis in the PRs, we performed colocalization studies of Vps26 and Rh1. In the dark, Rh1 and Vps26 signals do not overlap (Figure 5E). In contrast, numerous Rh1 punctae colocalize with Vps26 in the PR cell body upon transient light exposure (Figure 5E). These data indicate that endocytosed Rh1 is a cargo of the retromer.

### Reducing Endocytosis or Rh1 Levels Suppresses PR Degeneration in *Vps26* Mutants

**Figure 6:**
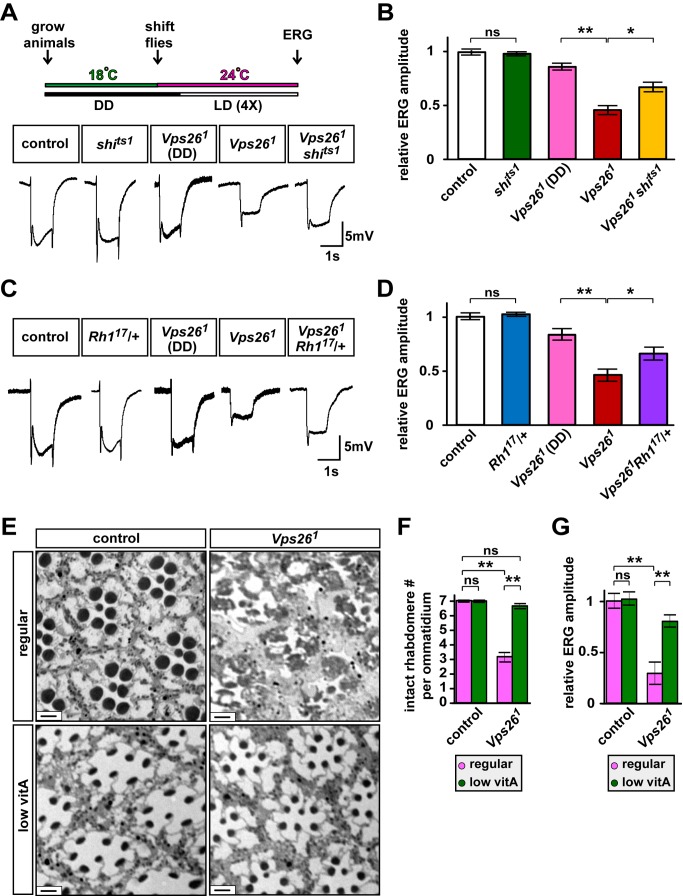
Decreasing Rh1 levels suppresses PR degeneration in *Vps26* mutants. (A) Decreasing endocytosis with a temperature-sensitive dynamin mutation (*shi^ts^*) suppresses ERG defects associated with the loss of Vps26. Animals were grown at 18°C in the dark. Newly eclosed flies were selected and kept in the dark for an additional 12 h at 24°C to reduce dynamin function in *shi^ts1^* or *Vps26^1^,shi^ts1^*. Subsequently, flies are shifted to LD or kept in DD at 24°C. ERG is performed at the end of the fourth LD cycle. *shi^ts1^* flies exhibit a normal ERG response. ERG defects in *Vps26^1^* mutant PRs are suppressed when dynamin function is partially reduced, as the *Vps26^1^,shi^ts1^* double mutants show a stronger depolarization than that in *Vps26^1^*. (B) Quantification of ERG amplitude of the flies tested in (A). Ten flies were measured for each genotype after completing the fourth light cycle. ERG amplitudes are normalized to control (iso). Student's *t* test; error bars represent SEM; * *p*<0.05; ** *p*<0.01; ns, no significance. (C) Reducing Rh1 suppresses ERG defects in *Vps26^1^* mutants. Heterozygous *Rh1^17^* mutants exhibit a normal ERG response. After 4 d in LD, loss of one copy of the *Rh1* gene strongly suppresses the reduction of ERG amplitude in *Vps26^1^* mutants. (D) Quantification of ERG amplitude of the flies tested in (C). Ten flies were recorded for each genotype at day 4. ERG amplitudes are normalized to control. Student's *t* test; error bars represent SEM; * *p*<0.05; ** *p*<0.01; ns, no significance. (E) Reducing dietary vitamin A (vitA) strongly suppresses PR degeneration in *Vps26^1^* mutants. Decreasing vitA lowers the levels of retinal, which is required for Rh1 maturation [39]. Loss of retinal therefore leads to loss of Rh1. Bright field images show that PR degeneration is strongly suppressed in *Vps26^1^* mutants raised on the low vitA–containing food when flies are kept in LD for 10 d. As vitA is a key component for Rh1 maturation and Rh1 is a key structural component of rhabdomeres, the rhabdomere size is much reduced when the flies were raised on low vitA food. Regular, regular fly food; low vitA, low vitamin A fly food. Scale bar, 2 µm. (F) Quantification of intact rhabdomere numbers in flies described in (E). Thirty ommatidia from three animals are examined for each genotype. Student's *t* test; error bars represent SEM. ** *p*<0.01; ns, no significance. (G) Quantification of ERG amplitudes of control and *Vps26^1^* raised on regular or low vitA fly food in LD after 10 d. Ten ERG traces were measured for corresponding genotypes and conditions. The amplitudes are normalized to control flies that are raised on regular food. Student's *t* test; error bars represent SEM; ** *p*<0.01; ns, no significance.

If PR degeneration in retromer mutants is caused by aberrant accumulation of Rh1 in the endolysosomal pathway, reducing the amount of endocytosed Rh1 or the abundance of Rh1 itself may suppress PR degenerative phenotypes. Dynamin (encoded by the *shi* gene) is required for Rh1 endocytosis upon light exposure [73]. *shi^ts1^*, a temperature-sensitive dynamin allele, encodes a mutant protein that is functional at 18°C but loses its function at elevated temperature [96]. Although impaired dynamin function leads to death of adult animals at 26°C or higher temperature (unpublished data), *shi^ts1^* mutant flies are viable and do not exhibit ERG defects at 24°C (Figure 6A–B). This permits us to conditionally reduce dynamin function in adult flies without impairing their light response. To address whether internalization of Rh1 is critical for the PR degeneration in *Vps26^1^* flies, we raised the *Vps26^1^*,*shi^ts1^* double mutants at 18°C and shifted the newly eclosed flies to 24°C to decrease dynamin function. We then performed ERGs on *Vps26^1^,shi^ts1^* double mutants kept in LD (Figure 6A). As shown in Figure 6A–B, reducing dynamin function significantly suppresses the loss in ERG amplitude in *Vps26^1^* mutants at day 4 in LD, indicating that Rh1 endocytosis plays an important role in PR degeneration upon loss of retromer function.

To address if a reduction in Rh1 suppresses the demise of the PRs, we tested flies that lack one copy of the *Rh1* gene. Strong hypomorphic or null *Rh1* alleles impair rhabdomere development in homozygous animals, as Rh1 plays a key signaling role in maintaining the structure of rhabdomeres [97],[98]. However, flies that are heterozygous for *Rh1* loss-of-function alleles do not show aberrant PR development even when the Rh1 protein levels are reduced by 50% (Figure S10 and [50]). To assess whether a reduction of 50% in Rh1 levels is able to suppress the *Vps26*-associated PR degenerative phenotypes, we tested ERGs in mutants that carry a single copy of the *Rh1* null allele (*ninaE^17^*, [99]). As shown in Figure 6C–D, flies that carry a single copy of *Rh1* exhibit a significant suppression of the ERG defects seen in *Vps26^1^* mutant flies that have been kept in LD for 4 d.

To strongly reduce Rh1 levels, we next raised *Vps26^1^* mutants on low vitamin A containing fly food. Vitamin A is a dietary precursor of 11-*cis* 3-hydroxyretinal, the chromophore that is covalently linked to the Rh1 opsin [100]. A decrease in vitamin A leads to loss of the chromophore. Because the chromophore is essential for Rh1 folding and stability [39],[101], absence of vitamin A leads to reduced Rh1 levels in flies. Although this manipulation still allows the formation of rhabdomeres, the size is dramatically reduced due to the requirement of Rh1 as a major structural component [102]. Indeed, a reduction in dietary vitamin A leads to diminished rhabdomere size in both control and *Vps26^1^* mutants. Remarkably, vitamin A deprivation leads to a very strong suppression of the PR degeneration observed in *Vps26^1^* mutants at day 10 in LD (Figure 6E–F). In addition, the reduced ERG amplitudes of *Vps26^1^* are strongly suppressed upon vitamin A deprivation (Figure 6G). In summary, decreasing Rh1 levels suppresses the retinal degeneration associated with *Vps26^1^*. This also suggests that elevated levels of Rh1 trapped in the endolysosomal pathway, rather than alterations in the levels of Rh1 in the rhabdomeres *per se*, underlie PR degeneration in *Vps26^1^* mutants.

### Increased Levels of Vps35 or Vps26 Are Sufficient to Suppress PR Degeneration

**Figure 7:**
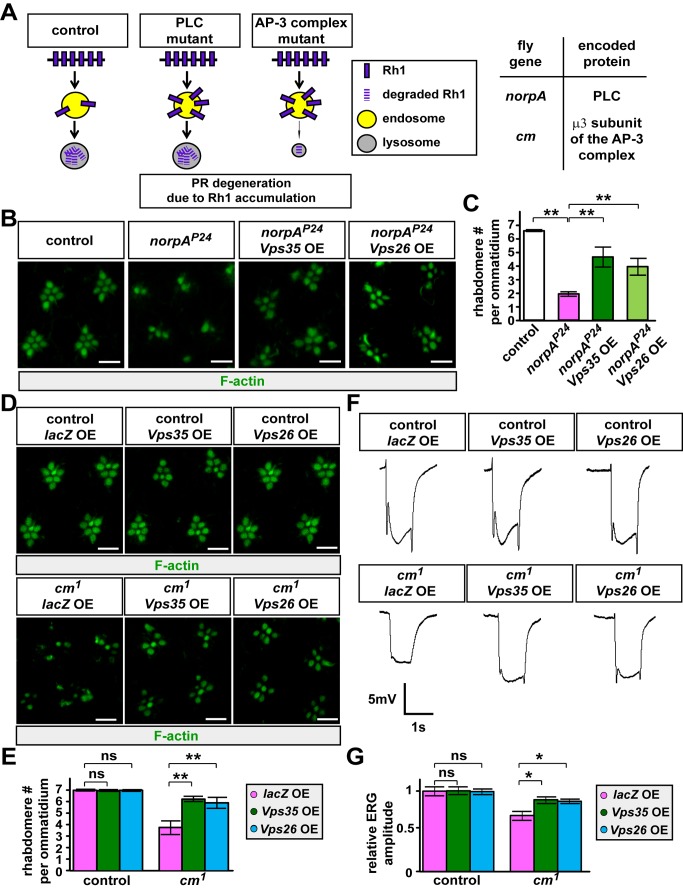
Increasing the levels of Vps35 or Vps26 suppresses PR degeneration associated with PLC and AP-μ3 mutants. (A) Schematic showing that mutations affecting PLC (*norpA*) or the μ3 subunit of the AP-3 complex (*cm*) lead to PR degeneration due to Rh1 accumulation in late endosomes. (B) Immunostaining for F-actin to mark rhabdomeres in the retina of control (*w^1118^*, left panel), *norpA^P24^* mutants (middle left panel), *norpA^P24^;actin-GAL>UAS-Vps35,UAS-w RNAi* (middle right panel), or *norpA^P24^;actin-GAL>UAS-Vps26,UAS-w RNAi* (right panel) flies upon 6 d of continuous exposure to whole-spectrum light. The number of rhabdomeres and organization of ommatidia of *norpA^P24^* mutants are severely reduced or impaired upon a 6-d exposure to light (middle left panel) but remain largely intact when Vps35 (middle right panel) or Vps26 (right panel) is overexpressed. OE, overexpression. Scale bar, 5 µm. (C) Quantification of the rhabdomere number per ommatidium for the indicated genotypes in (B). Sixty ommatidia from six animals were examined for each genotype. Student's *t* test; error bars represent SEM; ** *p*<0.01. (D) Immunostaining for F-actin to mark rhabdomeres in the retina of control flies overexpressing lacZ (*actin-GAL4>UAS-lacZ*, top left panel), Vps35 (*actin-GAL4>UAS-Vps35*, top middle panel), or Vps26 (*actin-GAL4>UAS-Vps26*, top right panel), or *cm^1^* mutants overexpressing lacZ (*cm^1^;actin-GAL4>UAS-lacZ*, bottom left panel), Vps35 (*cm^1^;actin-GAL4>UAS-Vps35*, bottom middle panel), or Vps26 (*cm^1^;actin-GAL4>UAS-Vps26*, bottom right panel) upon constant white light exposure for 6 d. The UAS-*w* RNAi is co-expressed in all animals. The organization and size of *cm^1^* mutant rhabdomeres are strongly impaired upon light exposure. Vps35 or Vps26 OE suppresses the PR morphological defects in *cm^1^* mutants. Scale bar, 5 µm. (E) Quantification of the rhabdomere number per ommatidium in the indicated genotypes. Sixty ommatidia from six animals were examined for each genotype. Student's *t* test; error bars represent SEM; ** *p*<0.01; ns, no significance. (F) ERG traces of control overexpressing lacZ (*actin-GAL4>UAS-lacZ*, top left panel), Vps35 (*actin-GAL4>UAS-Vps35*, top middle panel), or Vps26 (*actin-GAL4>UAS-Vps26*, top right panel), or *cm^1^* mutants overexpressing lacZ (*cm^1^;actin-GAL4>UAS-lacZ*, bottom left panel), Vps35 (*cm^1^;actin-GAL4>UAS-Vps35*, bottom middle panel), or Vps26 (*cm^1^;actin-GAL4>UAS-Vps26*, bottom right panel) flies. The *w* RNAi is co-expressed in each animal. After light exposure for 6 d, flies were transferred to the dark for 1 d to avoid visual adaptation to light. *cm^1^* mutants show a reduction of ERG amplitudes and loss of the on- and off-transients. Vps35 or Vps26 OE partially restores the on and offs and ERG amplitudes. (G) Quantification of ERG amplitudes of the flies indicated in (F). Ten ERG traces were recorded for each genotype. ERG amplitudes are normalized to control PRs. Student's *t* test; error bars represent SEM; * *p*<0.05; ns, no significance.

Rh1 accumulation in late endosomes causes PR degeneration in flies that lack PLC activity (encoded by the *no receptor potential A* (*norpA*) gene, [76],[103]) (Figure 7A). If the retromer retrieves Rh1 from endosomes, increasing retromer levels might alleviate PR degeneration in *norpA* flies. We therefore overexpressed the fly *Vps35* (*actin-GAL>UAS-Vps35,UAS-w RNAi*) or *Vps26* (*actin-GAL>UAS-Vps26,UAS-w RNAi*) in *norpA^P24^* mutants. Upon continuous exposure to white light for 6 d, *norpA^P24^* mutants exhibit reduced numbers of rhabdomeres compared to control flies (Figure 7B, left and middle left panels and Figure 7C). In addition, the ommatidia organization is often aberrant in *norpA^P24^* mutants (Figure 7B, middle left panel), indicating that the PRs are undergoing a progressive demise. Expression of Vps35 or Vps26 significantly restores rhabdomere numbers (Figure 7B, middle right and right panel and Figure 7C), suggesting that this manipulation is sufficient to suppress PR degeneration caused by Rh1 accumulation. In addition, expression of human Vps26B also suppresses the *norpA^P24^* phenotypes (unpublished data). These data indicate that rerouting some of the Rh1 slated for lysosomal degradation delays PR degeneration.

Mutations that impair lysosomal function can also lead to Rh1-induced PR degeneration [76]. We therefore assessed whether increasing Vps35 or Vps26 expression suppresses PR degeneration in mutants that affect a subunit of the AP-3 complex. AP-μ3, encoded by *carmine* (*cm*), is required for the proper biogenesis of lysosomal-related organelles [104]. Upon 6 d of light exposure, *cm^1^* mutant PRs showed degenerative features [76], including loss of rhabdomeres and disruption of the ommatidia organization (Figure 7D, bottom left panel and Figure 7E). In addition, *cm^1^* mutant PRs exhibit decreased ERG amplitudes (Figure 7F–G). Similar to *norpA^P24^* mutants, increasing Vps35 or Vps26 in *cm^1^* mutants restores the number of rhabdomeres (Figure 7D–E) and suppresses ERG defects (Figure 7F–G). These data indicate that increased retromer function can not only prevent PR degeneration in mutants with impaired late endosomal trafficking but also suppress PR degeneration caused by defective lysosomal function.

### Vertebrate Retromer Homologs

**Figure 8:**
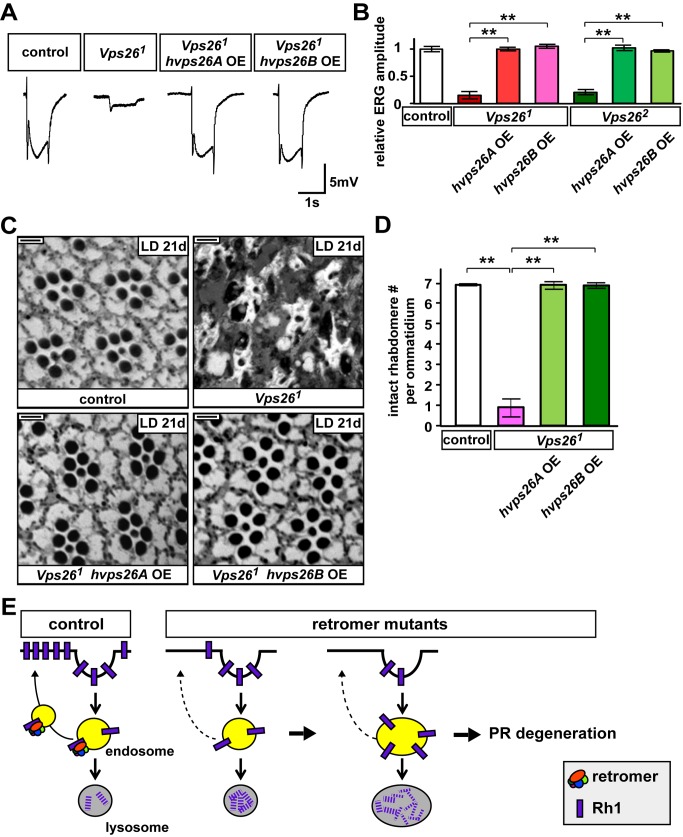
Human Vps26 homologs can rescue ERG loss in fly *Vps26* mutants. (A) ERG traces of control (iso), *Vps26^1^*, or *Vps26^1^* mutants that ubiquitously express human Vps26A (*Vps26^1^;actin-GAL4>UAS-hvps26A,UAS-w RNAi*) or Vps26B (*Vps26^1^;actin-GAL4>UAS-hvps26B,UAS-w RNAi*). Flies were kept in LD for 21 d. Both human Vps26A and Vps26B rescue the loss of on- and off-transients and reduced amplitudes of the ERGs in *Vps26^1^* mutant PRs. (B) Quantification of the amplitudes of the ERGs in flies in LD for 21 d. ERG amplitudes were normalized to control PRs. Student's *t* test; error bars represent SEM; ** *p*<0.01. (C) Bright field images of control, *Vps26^1^*, or *Vps26^1^* mutants that overexpress human *vps26A or vps26B* cDNA. The UAS-*w* RNAi is co-expressed with human *vps26A* or *vps26B* in the *Vps26^1^* mutants. Flies were kept in LD for 21 d. The PRs of *Vps26^1^* are severely impaired after 21 d in LD but remain intact when human Vps26 homologues are overexpressed. Scale bar, 2 µm. (D) Quantification of intact rhabdomere numbers in (C). Thirty ommatidia from three animals were examined for each genotype. Student's *t* test; error bars represent SEM; ** *p*<0.01. (E) Model: the retromer recycles Rh1 in fly PRs. In the absence of the retromer, endocytosed Rh1 is trapped in the endolysosomal compartments upon a short-term light exposure and an increased amount of endocytosed Rh1 is degraded. However, upon chronic light exposure, persistent Rh1 accumulation leads to an expansion of late endosomes and lysosomes. Imbalanced Rh1 homeostasis in the endolysosomal pathway in turn stresses PR cells, causing PR degeneration.

Because the genes that encode the retromer proteins are evolutionarily conserved (Figure 1A, [7]–[9]), we investigated whether the vertebrate Vps26 and Vps35 may also function in the visual system. As shown in Figure S11A, *vps26A* is expressed in the mouse retina based on Northern blots. In addition, we stained the retina of wild-type control or heterozygous mice containing a *lacZ* inserted in the *vps35* gene [105]. Most of the melanopsin-expressing ipRGCs (92%) also express β-GAL (Figure S11B). To test whether human Vps26 homologs can substitute for the function of fly Vps26 in PRs, we ubiquitously expressed the human *vps26A* or *vps26B* cDNA in the *Vps26^1^* mutant background. Both human Vps26A and Vps26B were able to rescue the lethality of *Vps26^1^* mutant flies (Figure 8A–D). We also recorded ERGs from adult flies kept in LD for 21 d. As shown in Figure 8A–B, we observed a full rescue of the loss of ERG amplitude and on–off transients. In addition, PR degeneration in *Vps26^1^* mutants is fully suppressed by overexpressing human Vps26 proteins (Figure 8C–D). These results show that the human Vps26 homologs are able to perform the same function as the fly Vps26 protein.

## Discussion

### The Retromer Complex Is Required for Maintenance of Fly PRs

The retromer recycles specific proteins from endosomes to the TGN or plasma membrane [7]–[9]. Here we show that, although the loss of the retromer does not obviously affect eye development, mutations in *Vps26* or *Vps35* genes lead to strong light-dependent PR degeneration (Figure 1B–E, Figure 2B–E, and Figure S1C–E). The demise of *Vps26^1^* and *Vps35^MH20^* PRs is associated with a significant increase in the number of late endosomes and lysosomes upon light exposure (Figure 3 and Figure S5), showing that the endolysosomal pathway is strongly affected when Rh1 recycling by the retromer is impaired. Indeed, Rh1 accumulates in late endosomes or lysosomes in the mutant PRs (Figure 4B–C). Although Rh1 can be degraded and the function of *Vps26^1^* mutant PRs is not abolished upon short light exposure (Figure 5A–D), chronic exposure to light is detrimental to *Vps26^1^* mutants because persistent Rh1 accumulation in the endolysosomal pathway is toxic to PR cells [71],[75],[76]. Hence, reducing Rh1 endocytosis or Rh1 levels in the rhabdomeres suppresses PR degeneration upon prolonged light exposure (Figure 6). Interestingly, increasing Vps35 or Vps26 in mutants that show PR degeneration due to Rh1 accumulation in the endolysosomal compartments [76] suppresses the degenerative phenotypes (Figure 7). In summary, the retromer is required to retrieve Rh1 from endosomes to maintain PR function and integrity (Figure 8E).

How does Rh1 internalization affect the endolysosomal pathway in retromer mutants? One possibility is that lysosomes in the mutants are unable to cope with the increased levels of internalized Rh1 over time as Rh1 is one of the most abundant proteins in PRs [106],[107]. This in turn triggers an increase in the number of lysosomes (Figures 3B,D,F and Figure S5B–C,E), the accumulation of aberrant lysosomes (Figure S5C), and the accumulation of endolysosomal intermediates, including late endosomes (Figure 3B,D,F and Figure S5B,D). Alternatively, loss of the retromer may increase the flux in the endolysosomal pathway, which overpowers the rate of endolysosomal maturation and leads to an adaptive response that eventually leads to the expansion of these compartments. Both pathways can lead to an apparent accumulation of Rh1 in the cell body when stained and analyzed by fluorescence microscopy (Figure 4 and Figure S9).

Defective regulation of Rh1 can lead to the demise of PRs via apoptosis [72],[108]. Does apoptosis play a critical role in the PR degeneration in retromer mutants? We argue that this is not the case based on the following observations. First, the retromer mutants exhibit progressive PR degeneration over a 3-wk period (Figure 1B–C, Figure 2B–C, and Figure S1C–E), whereas apoptosis typically occurs within hours [109]. Second, mutants that lead to PR loss via apoptosis lose most PRs within ommatidia by the engulfment of surrounding glial cells [110]. However, the degenerating *Vps26* and *Vps35* mutant PRs are not removed, although their morphology is very severely disrupted. Indeed, they can still be identified in 3-wk-old flies (Figure 1D, Figure 2D, Figure 8C, and Figure S1D), indicating a lack of engulfment by surrounding cells [111]. Third, overexpressing p35, a pan-caspase inhibitor of apoptosis [108], fails to suppress the PR degeneration in *Vps26* mutants upon light exposure (unpublished data). Indeed, Rh1 accumulation in different subcellular compartments triggers different cellular responses, which leads to PR degeneration of varying severity [2]. Accumulation of Rh1 in the endolysosomal pathway seems particularly toxic to PRs but often does not cause the removal of PRs for many weeks.

Loss of Wg affects eye development [112], whereas loss of Crumbs leads to short and/or fused rhabdomeres [113],[114]. Although the retromer can recycle Wls or Crumbs in some fly tissues [10]–[14],[17],[18], *Vps26^1^* or *Vps35^MH20^* mutants do not exhibit obvious eye developmental defects. It is therefore very likely that the composition of the eye retromer is different from the wing retromer. Indeed, loss of Snx3 does not cause obvious degenerative phenotypes when compared to loss of Vps26 or Vps35 (compare Figure S3 with Figures 1 and 2), yet these proteins are all essential for the recycling of Wls and wing development [10]–[14].

### The Retromer in Photosensitive Cells in Flies and Vertebrates

Many players required for phototransduction in *Drosophila* are conserved in a phototransduction cascade in vertebrate ipRGCs. These include melanopsin, PLC, and the TRP channels [60]–[63]. This phototransduction pathway plays a role in photoentrainment of circadian rhythms [64]–[67] and the control of the pupillary light reflex [64],[68],[69]. We find that *vps35* is expressed in 92% of the melanopsin-expressing RGCs in the mouse retina (Figure S11B). This may be an underestimate due to technical difficulties with β-GAL immunostaining in mouse tissues. In addition, the human Vps26 proteins are able to substitute for the function of the fly homolog in PRs (Figure 8A–D). Because vertebrate melanopsin has very similar photochemical properties to fly rhodopsin [115],[116], the retromer may play a conserved role in vertebrate ipRGCs. As deletion of *vps35* leads to early embryonic lethality [105], a *vps35* conditional KO in ipRGCs will need to be established to address its role in ipRGCs.

### Implications of the Retromer Complex in Human Neurodegenerative Diseases

The retromer has been implicated in human neurodegenerative disease, including Alzheimer's disease (AD) and PD [19],[20],[117]. In AD, a retromer deficiency has been proposed to affect subcellular distribution of β-secretase, which leads to increased amyloid-beta (Aβ) deposits and defective neuronal function [105],[118]. In PD, a missense mutation in *VPS35* (D620N) has been shown to cause an autosomal dominant late onset form of the disease [19],[20]. The Vps35 D620N mutant protein appears to function as a dominant negative, and Vps35 and LRRK2 (Leucine-Rich Repeat Kinase 2) have been shown to interact [119]. It will therefore be interesting to assess if these mutants affect ipRGCs as PD patients often have sleep issues [120].

## Materials and Methods

### Fly Strains and Genetics

*Vps26^1^* and *Vps26^2^* alleles were isolated as described [6],[78]. Male large duplications (∼1–2 Mb) covering the X chromosome [121] were crossed with female y,w,mut*,P{neoFRT}19A^isogenized^ flies that were balanced with *FM7c,Kr-GAL4,UAS-GFP*(*Kr>GFP*). For the *XE52* group, the lethality of alleles was rescued by *Dp(1;Y)y^2^67g19.2* (1A1;2B17-18 + 20A3;20Fh). *XE52* alleles complemented all the available deficiencies covered by *Dp(1;Y)y^2^67g19.2* [122],[123]. Therefore, we turned to cross the *XE52* alleles with 80 kb P[acman] duplications that cover the gaps among deficiencies [124] and found three overlapping duplications (*Dp(1;3)DC446*, *Dp(1;3)DC033*, and *Dp(1;3)DC034*) that rescued the lethality of the mutants. These P[acman] duplications share a ∼21 kb interval. We then performed PCR sequencing for genes localized to this region and identified mutations in *Vps26*. A lethal insertion *P{EP}Vps26^G2008^* in *Vps26* failed to complement both *XE52* alleles.

*Rh1^17^* [84], *shi^ts1^* [81], *y,w,GMR-hid,cl,P{neoFRT}19A* [125], *norpA^P24^* [65], *cm^1^* [89], *w^1118^* [126], *UAS*-*w* RNAi [127], and *GMR-w-RNAi^13D^* [128] were obtained from the Bloomington Drosophila Stock Center (BDSC). *Vps35^MH20^* is a gift from John-Paul Vincent [13]; *Snx1^d1^* and *Snx^d3^* are gifts from Xinhua Lin [10]; *UAS*-*Lamp1::GFP* is a gift from Helmut Krämer [95]; and the UAS-*GFP* RNAi is a gift from Norbert Perrimon [129]. UAS-RNAi stocks against the *wash* or *wls* genes were obtained from the Vienna Drosophila RNAi Center (VDRC). *shi^ts1^* was used to generate the *y,w,Vps26^1^,shi^ts1^,P{neoFRT}19A* recombinant, whereas the *GMR-w-RNAi^13D^* was used to generate the *y,w,GMR-w-RNAi^13D^,P{neoFRT}19A* and *y,w,Vps26^1^,GMR-w-RNAi^13D^,P{neoFRT}19A* recombinants.

For ERG, bright field, and TEM experiments, *y,w,Vps26^1^,P{neoFRT}19A/FM7c*, *Kr>GFP*, or *y,w,Vps26^2^,P{neoFRT}19A/FM7c,Kr>GFP* females were crossed with *cl(1),P{neoFRT}19A/Dp(1;Y),y^+^,v^+^* [130]; *ey-FLP* males, whereas *w ; Vps35^MH20^,P{neoFRT}42D/CyO* females were crossed with *y,w,ey-FLP,GMR-lacZ ; w^+^,cl,P{neoFRT}42D* males. *w; Snx1^d1^, P{neoFRT}FRT40A* and *w; Snx3^d1^, P{neoFRT}FRT82B* mutants were crossed with *y,w,ey-FLP,GMR-lacZ; w^+^,cl,P{neoFRT}40A* and *y,w,ey-FLP,GMR-lacZ; w^+^,cl, P{neoFRT}82B*, respectively. White patches indicate mutant clones. Flies in which at least 90% of the eyes are mutant were examined. To produce clones for controls, homozygous iso were crossed with *cl(1),P{neoFRT}19A/Dp(1;Y)y^+^,v^+^; ey-FLP*; *y,w; P{neoFRT}42D* were crossed with *y,w,ey-FLP,GMR-lacZ; w^+^,cl,P{neoFRT}42D*; and *y,w; P{neoFRT}82B* were crossed with *y,w,ey-FLP; w^+^,cl,P{neoFRT}82B*. All the flies were raised in constant darkness before experiments. For immunostaining in wing imaginal discs of third instar larvae, *y,w,Vps26^1^,P{neoFRT}19A/Dp(1;Y)901* males were crossed with *ubi-GFP,cl,FRT19A ; hh-GAL4,UAS-FLP* [13] females to generate large mutant clones in the posterior region. In eye imaginal discs of third instar larvae, *w; Vps35^MH20^,P{neoFRT}42D/CyO,Kr>GFP* [13] females were crossed with *y,w,ey-FLP,GMR-lacZ; ubi-GFP,cl(2),P{neoFRT}42D*.

For immunostaining, we crossed iso or *y,w,Vps26^1^,P{neoFRT}19A/Dp(1;Y)901* males with *y,w,GMR-hid,cl,P{neoFRT}19A; ey-FLP* females to remove *w^+^* wild-type cells in the eyes. To generate *Vps26^1^* clones that express Lamp1::GFP, we crossed the *y,w,Vps26^1^,GMR-w-RNAi^13D^,P{neoFRT}19A; actin-GAL4>UAS-Lamp1::GFP* females with *cl(1),P{neoFRT}19A/Dp(1;Y),y^+^,v^+^* [130]; *ey-FLP* males to remove eye pigments that are generated by the mini-w^+^ markers in the *actin-GAL4* and *UAS-Lamp1::GFP* transgenic constructs.

Crosses were kept in the dark to prevent flies from being exposed to light before experiments. The newly eclosed flies (1 d after eclosion) were used for all the experiments.

Following are the genotypes used in our analyses: controls — *y,w,P{neoFRT}19A/cl(1),P{neoFRT}19A; ey-FLP/+* ( = iso in Figures 1B–E, 3A, 6, 8A–D, S1B, S5A, S6A, S8, and S10); *y,w,P{neoFRT}19A/y,GMR-hid,cl,P{neoFRT}19A; ey-FLP/+* ( = iso in Figure 4A, Figure 4B, top left panel, Figure 4C, top panel, Figure 5A–B,E, Figure S7, and Figure S9); *y,w,ey-FLP,GMR-lacZ/w; P{neoFRT}42D,Vps35^MH20^/P{neoFRT}42D,ubi-GFP,cl* (GFP positive tissues in Figure 2A); *y,w,ey-FLP,GMR-lacZ/w; P{neoFRT}42D/P{neoFRT}42D,w^+^,cl* ( = *FRT42D* in Figure 2B–E); *y,w,GMR-w-RNAi^13D^,P{neoFRT}19A/cl(1),P{neoFRT}19A; ey-FLP/actin-GAL4>UAS-Lamp1::GFP* ( = iso in Figure 4B, bottom left panel and Figure 4C, bottom panel); *w^1118^* (Figure 7B–C); *w; actin-GAL4/UAS-w RNAi* (Figure 7D–G); *y,w,Vps26^1^,P{neoFRT}19A/ubi-GFP,cl,P{neoFRT}19A; hh-GAL4>UAS-FLP/+* (GFP positive tissues in Figure S2C–D); *y,w,P{neoFRT}19A* ( = iso in Figure S2F–G); *y,w,ey-FLP,GMR-lacZ/y,w; P{neoFRT}82B/P{neoFRT}82B,w^+^,cl* ( = *FRT82B* in Figure S3A–B); *GMR-GAL4>UAS-w RNAi/UAS-GFP RNAi* ( = *GMR>GFP RNAi* in Figure S4A–C); *Rh1-GAL4/UAS-GFP RNAi* ( = *Rh1>GFP RNAi* in Figure S4D–E). Generation of *Vps26* mutant clones — *y,w,Vps26^1^,P{neoFRT}19A/cl(1),P{neoFRT}19A; ey-FLP/+* ( = *Vps26^1^* in Figures 1B–E, 3B–C, 6, 8A–D, S1B, S5B–C, S6B–C, and S10); *y,w,Vps26^2^,P{neoFRT}19A/cl(1),P{neoFRT}19A; ey-FLP/+* ( = *Vps26^2^* in Figures 1C and S1B–E); *y,w,Vps26^1^,P{neoFRT}19A/y,GMR-hid,cl,P{neoFRT}19A; ey-FLP/+* ( = *Vps26^1^* in Figure 4A, Figure 4B, top right panel, Figure 4C, top panel, Figure 5A–D, and Figure S9); *y,w,Vps26^1^,GMR-w-RNAi^13D^;P{neoFRT}19A/cl(1),P{neoFRT}19A; ey-FLP/actin-GAL4>UAS-Lamp1::GFP* ( = *Vps26^1^* in Figure 4B, bottom right panel and Figure 4D, bottom panel); *y,w,Vps26^1^,shi^ts1^,P{neoFRT}19A/cl(1),P{neoFRT}19A; ey-FLP/+* ( = *Vps26^1^*,*shi^ts1^* in Figure 6A–B); *y,w,Vps26^1^,P{neoFRT}19A/cl(1),P{neoFRT}19A; ey-FLP; Rh1^17^/+* ( = *Vps26^1^; Rh1^17^/+* in Figure 6C–D and Figure S10); *y,w,Vps26^1^,P{neoFRT}19A/ubi-GFP,cl,P{neoFRT}19A; hh-GAL4>UAS-FLP/+* (GFP negative tissues in Figure S2C–D). Generation of *Vps35* mutant clones — *y,w,ey-FLP,GMR-lacZ/w; P{neoFRT}42D,Vps35^MH20^/P{neoFRT}42D,ubi-GFP,cl* (GFP negative tissues in Figure 2A); *y,w,ey-FLP,GMR-lacZ/w; P{neoFRT}42D,Vps35^MH20^/P{neoFRT}42D,w^+^,cl* ( = *Vps35^MH20^* in Figures 2B–E, 3D–E, S5D–E, and S6D–E). Generation *Snx1* or *Snx3* mutant clones in the eyes — *y,w,ey-FLP,GMR-lacZ/w; P{neoFRT}40A,Snx1^d1^/P{neoFRT}40A,w^+^,cl* ( = *Snx1^d1^* in Figure S3); *y,w,ey-FLP,GMR-lacZ/w; P{neoFRT}82B,Snx3^d1^/P{neoFRT}82B,w+,cl* ( = *Snx3^d1^* in Figure S3). Rescued *Vps26* mutants — *y,w,Vps26^1^,P{neoFRT}19A/Y; Vps26-gEGFP/actin-GAL4>UAS-w RNAi* ( = *Vps26^1^* GR in Figure 1B–E); *y,w,Vps26^2^,P{neoFRT}19A/Y; Vps26-gEGFP/actin-GAL4>UAS-w RNAi* ( = *Vps26^2^* GR in Figure 1C and Figure S1C–E); *y,w,Vps26^1^,P{neoFRT}19A/Y; actin-GAL4>UAS-w RNAi/UAS-hvps26A* ( = *Vps26^1^ hvps26A* OE in Figure 8A–D); *y,w,Vps26^1^,P{neoFRT}19A/Y; actin-GAL4>UAS-w RNAi/UAS-hvps26B* ( = *Vps26^1^ hvps26B* OE in Figure 8A–D); *y,w,Vps26^1^,P{neoFRT}19A/Y; Vps26-gEGFP/+* (Figure S2B); *y,w,Vps26^2^,P{neoFRT}19A/Y; Vps26-gEGFP/+* (Figure S2B); *y,w,Vps26^1^,P{neoFRT}19A/Y; tub-GAL4>UAS-Vps26* (Figure S2B); *y,w,Vps26^2^,P{neoFRT}19A/Y; tub-GAL4>UAS-Vps26* (Figure S2B). Overexpression of LacZ, Vps35, or Vps26 — *w,norpA^P24^/Y; actin-GAL4>UAS-w RNAi/UAS-Vps35* ( = *norpA^P24^ Vps35* OE in Figure 7B–C); *w,norpA^P24^/Y; actin-GAL4>UAS-w RNAi/UAS-Vps26* ( = *norpA^P24^ Vps26* OE in Figure 7B–C); *w; actin-GAL4>UAS-w RNAi/UAS-lacZ* ( = control *lacZ* OE in Figure 7D–G); *w; actin-GAL4>UAS-w RNAi/UAS-Vps35* ( = control *Vps35* OE in Figure 7D–G); *w; actin-GAL4>UAS-wRNAi/UAS-Vps26* ( = control *Vps26* OE in Figure 7D–G); *cm^1^/Y; actin-GAL4>UAS-w RNAi/UAS-lacZ* ( = *cm^1^ lacZ* OE in Figure 7D–G); *cm^1^/Y; actin-GAL4>UAS-w RNAi/UAS-Vps35* ( = *cm^1^ Vps35* OE in Figure 7D–G); *cm^1^/Y; actin-GAL4>UAS-w RNAi/UAS-Vps26* ( = *cm^1^ Vps26* OE in Figure 7D–G). RNAi knockdown of *wash* — *w; GMR-GAL4>UAS-w RNAi/UAS-wash RNAi* ( = *GMR>wash RNAi* in Figure S4A–C); *w; Rh1-GAL4/UAS-wash RNAi* ( = *Rh1>wash RNAi* in Figure S4D–E). Miscellaneous — *w,shi^ts1^* ( = *shi^ts1^* in Figure 6A–B); *w; Rh1^17^/+* ( = *Rh1^17^/+* in Figure 6C–D and Figure S10); *w,norpA^P24^* ( = *norpA^P24^* in Figure 7B–C); *w,P{EP}Vps26^G2008^/Y* ( = *P{EP}Vps26^G2008^* in Figure S2B).

### Immunostaining of Fly Tissues

For whole mount staining, we dissected fly tissue samples in ice cold PBS and fixed them with 4% paraformaldehyde at room temperature for 30 min as described [6]. For tissue sections, we fixed fly tissues with 4% paraformaldehyde, dehydrated the tissues with acetone, and embedded them in LR white resin (Electron Microscopy Sciences). Images were captured with a Zeiss LSM 710 confocal microscope. Antibodies were used at the following concentrations: rabbit anti-Rab5 (abcam), 1∶500; rabbit anti-Avl [84], 1∶500; rabbit anti-Rab7 [76], 1∶500; mouse anti-PDI (abcam), 1∶100; rabbit anti-GM130 (abcam), 1∶500; rabbit anti-Rab11 (abcam, [6]), 1∶500; chicken anti-GFP (abcam), 1∶1,000; mouse anti-Rh1 [4C5, Developmental Studies Hybridoma Bank (DSHB)], 1∶50; mouse anti-Wash (P3H3, [131]), 1∶20; guinea pig anti-Vps26, 1∶1,000 (this study); biotin-conjugated PNA (Vector Labs), 1∶1,000; Alexa 488-conjugated phalloidin (Invitrogen), 1∶100; and Alexa 405-, Alexa 488-, Cy3-, or Cy5-conjugated secondary antibodies (Jackson ImmunoResearch), 1∶600.

### Western Blotting

Fly heads were homogenized in 1× Laemmli sample buffer (Bio-Rad) containing 2.5% β-mercaptoethanol (Sigma-Aldrich). Tissue samples were loaded into 10% gels, separated by SDS-PAGE, and transferred to nitrocellulose membranes (Bio-Rad). Antibodies were as follows: mouse anti-actin (ICN Biomedicals), 1∶2,500; mouse anti-Rh1 (4C5, DSHB), 1∶1,000; and rabbit anti-TRP [58].

### Molecular Biology

#### Cloning

Primers 5′-CTTCGGCCGGCCACCATGAATTTCCTGGGATTC-3′ and 5′-GCTTCTAGATTAGGTGGAGTCCAGGCCCAGCAGGGGGTTGGGGATGGGCTTGCCATCGGTGGCCAACGGCGTTG-3′ were used to clone the full-length fly *Vps26* cDNA from a plasmid (LD29140) obtained from the Berkeley Drosophila Genome Project (BDGP). A C′-terminal V5 tag was designed in-frame with *Vps26*. The *Vps26* PCR product and the pUAST-attB vector were digested with EagI (NEB) and XbaI (NEB). T4 ligase (NEB) was used to ligate the fly *Vps26* PCR products with pUAST-attB vector. Full-length fly Vps35 cDNA was cloned from a plasmid (SD03023 from BDGP) using primers 5′-CTTCTCGAGGCCACCATGTATCCCTGGAGCTCC-3′ and 5′-TTCTCTAGATTAAGCGTAGTCTGGGACGTCGTATGGGTAATTGAGAGTTATGCCCG-3′. A C-terminal HA tag was added to *Vps35*. *Vps35* PCR product was digested with XhoI (NEB) and XbaI (NEB) and ligated with the pUAST-attB vector. Full-length human *vps26A* and *vps26B* were cloned from plasmids (HsCD00296497 and HsCD00076427, respectively) (Biodesign Institute of Arizona State University). The following primers were used for PCR cloning: for *hvps26A*, 5′-GTTGAATTCGCCACCATGAGTTTTCTTGGAGGCTT-3′ and 5′-GCATCTAGATTAAGCGTAGTCTGGGACGTCGTATGGGTACATTTCAGGCTGTTCGGCAG-3′; for *hvps26B*, 5′-GTTGAATTCGCCACCATGAGCTTCTTCGGCTTCGG-3′ and 5′-GAATCTAGATTAAGCGTAGTCTGGGACGTCGTATGGGTACTGCCTGCAGTTGTTGTCAG-3′. A C-terminal HA tag was added to human *vps26* isoforms. PCR products were digested with EcoRI (NEB) and XbaI (NEB) and ligated with pUAST-attB vectors. Fly *Vps26::V5* and *Vps35::HA* and human *vps26A::HA* and *vps26B::HA* cDNA constructs were purified and injected into *y,w,ΦC31 ; VK33* (fly cDNAs) or *y,w,ΦC31 ; VK37* embryos (human cDNAs), and the transgenic flies were selected.

#### Genomic tagging

Recombineering was performed to tag *EGFP* at the C-terminus of the *Vps26* gene. We used primers 5′-GCAATTACGCCCAATACGAAAAAGACCGGTGCAACGCCGTTGGCCACCGATGCAGCCCAATTCCGATCATATTC-3′ and 5′-GTTACTACTTATCCCCCTGAAGATGGAAATCCTCTCCAGCTTCCCACTCATTACTTGTACAGCTCGTCCATG-3′ to amplify a PCR product containing the EGFP tag from the PL452-EGFP template vector [132]. We then electroporated the EGFP-tagged PCR products into DY380 bacteria, which had been previously transformed with a 20 kb P[acman] genomic rescue construct that covers the entire *Vps26* genomic locus (clone CH322-92A18 from the BACPAC Resources Center, [133]). Recombineering and related procedures were performed according to the standard protocol [132]. *Vps26-gEGFP*–tagged genomic construct was injected into *y,w,ΦC31 ; VK31* embryos, and the transgenic flies were selected.

### Generation of Anti-Fly Vps26 Antibody

Full-length fly *Vps26* cDNA was cloned from LD29140 by PCR with primers 5′-CTTGGATCCATGAATTTCCTGGGATTCGGCCA-3′ and 5′-CTGGCGGCCGCTCAATCGGTGGCCAACGGC-3′. PCR products were then digested with BamHI (NEB) and NotI (NEB) and ligated with a pET28a(+) vector containing a Hisx6 tag at the N′-terminus (Novagen). The Vps26-pET28a(+) construct was transformed into BL21(DE3)pLysS bacteria (Invitrogen). Expression of Hisx6-Vps26 was induced with 0.5 mM isopropylthio-β-galactoside (IPTG, Sigma-Aldrich), and the recombinant protein was purified with a Ni-NTA column (Qiagen) and inoculated into guinea pigs to generate polyclonal antibodies (Cocalico Biological Inc.).

### Light/Dark Cycle and Blue-Orange Light Exposure

In general, mosaic flies were grown in the dark at 25°C, and the newly eclosed flies were selected and shifted to a 12-h light/dark cycle (LD, 1,800 lux in the light cycle) or constant darkness (DD) at 25°C for the indicated periods. In the *shi^ts^* suppressor assay, animals were raised in the dark at 18°C to avoid reduced dynamin protein function. Newly eclosed flies were then shifted to 24°C in the dark for 12 h. Flies were then kept in LD or DD at 24°C. For aging experiments, flies were flipped to new vials every 3 d to maintain food conditions, and the positions of the vials were randomly shuffled to normalize light exposure. For pulse-chase assays, flies were kept in a box with a blue LED [6] for 20 min and then an orange LED for 5 min.

### Low Vitamin A–Containing Food

Low vitamin A–containing food was made according to [134]. In brief, a solution containing 10% dry yeast, 10% sucrose, 0.02% cholesterol, and 2% agar was microwaved and poured into fly vials. After drying at room temperature overnight, fresh low vitamin A food was used for experiments.

### TEM and Bright Field Imaging

To prepare fly retina samples for TEM and bright field imaging, fly heads were dissected and fixed at 4°C in 4% paraformaldehyde, 2% glutaraldehyde, 0.1 M sodium cacodylate, and 0.005% CaCl_2_ (PH 7.2) overnight; postfixed in 1% OsO_4_; dehydrated in ethanol and propylene oxide; and then embedded in Embed-812 resin (Electron Microscopy Sciences) with vacuum attachments. PRs were then sectioned and stained in 4% uranyl acetate and 2.5% lead nitrate. TEM images of PR sections were taken using a JEOL JEM 1010 transmission electron microscope with an AMT XR-16 mid-mount 16 mega-pixel digital camera, while bright field pictures were captured using the Zeiss Imager.Z2 light microscope with an AxioCamMRm digital camera. The morphology and quantity of rhabdomeres, endosomes, and lysosomes were determined in Image J.

### ERGs

ERG recordings were performed as described [6]. Briefly, adult flies were glued to a glass slide, a recording probe was placed on the surface of the eye, and a reference probe was inserted in the thorax. A 1-s flash of white light was given, and the response was recorded and analyzed using the AXON™-pCLAMP^®^8 software.

### Mouse Retina cDNA Preparation and Northern Blot Analysis

Mouse retina cDNA was prepared as described [135]. *vps26A* was PCR-amplified using primers 5′-GAGTTTTCTTGGAGGCTTTTTTGGTCC-3′ and 5′-TTACATCTCAGGCTGCTCCGCAGAGG-3′ and a 30 ng cDNA template. The PCR product was cloned into pBluescript by blunt-end ligation and verified by sequencing. DNA probes were generated from gel-purified insert (excised with EcoRI and HindIII) by random-prime labeling with [α-^32^P]dCTP using the DECAprimeII kit (Ambion). Unincorporated nucleotides were removed with a MicroBio-Spin 30 column (Bio-Rad).

For Northern blot analysis, total RNA was prepared from retinas of 3–4-mo-old CD-1 mice by homogenization in TRI reagent (Ambion) using a motorized pestle and passage through a 20-gauge needle, extraction with 1-bromo-3-chloro-propane, and purification with the RNeasy kit (Qiagen). We resolved 10 µg of total RNA on a formaldehyde-agarose gel and transferred it to a Hybond-N+ nylon membrane (GE Healthcare) by capillary transfer. Membranes were prehybridized in ULTRAhyb (Ambion) for 1 h, then incubated at 42°C overnight with a ∼2.5×10^7^ cpm denatured probe diluted in ULTRAhyb. Blots were washed several times in 2× SSC+0.1% SDS and several times in 0.1× SSC+0.1% SDS. Washes were performed at 42–45°C. For imaging, blots were exposed to a storage phosphor screen and scanned on a Typhoon (GE Healthcare).

### β-Gal and Melanopsin Detection in Mouse Retina

For immunohistochemistry, wild-type or *vps35 lacZ* KI [105] mouse eyes were fixed in 4% paraformaldehyde in PBS for 45 min, washed in PBS, then stored overnight in 30% sucrose in PBS. The cornea and lens were removed, and eyecups were embedded in OCT (Tissue-Tek) and flash-frozen. Cryosectioned retina were fixed in 2% paraformaldehyde in 1× PBS for 10 min and stained with the indicated antibodies: chicken anti–β-GAL (abcam, 1∶1,000) and rabbit α-melanopsin (Advanced Targeting Systems, 1∶5,000). Alexa 488- or Cy3-conjugated secondary antibodies (Jackson ImmunoResearch, 1∶600) were used for immunostaining. Images were captured using the Zeiss LSM 710 confocal microscope and analyzed using the Image J software.

### Statistical Analysis

Two-tailed Student's *t* test was used to analyze experimental results.

## Supporting Information

Figure S1 *Vps26^2^* mutants exhibit light-dependent PR degeneration. (A) Schematic of the phototransduction pathway in *Drosophila*. IP_3_, inositol 1,4,5-trisphosphate; PUFA, polyunsaturated fatty acids; BL, blue light; OL, orange light. (B) Adult mosaic eyes of control (iso), *Vps26^1^*, and *Vps26^2^* mosaic mutant eyes generated by *ey-FLP* in a cell lethal (*cl*) background. White regions mark mutant tissues in the *Vps26* alleles. Similar to control, *Vps26* mutants exhibit large clones with normal gross eye morphology. (C) ERG traces of *Vps26^2^* mosaic eyes at day 0, kept in LD (light intensity = 1,800 lux) for 2, 10, and 21 d; *Vps26^2^* rescued by the *Vps26-gEGFP* genomic rescue construct kept in LD for 21 d; and *Vps26^2^* kept in DD for 21 d. The UAS-*w* RNAi is expressed in the rescued animals. On- and off-transients disappear and the amplitude decreases upon 2 d in LD, but remain unchanged upon keeping the flies in DD for 3 wk. The loss of on- and off-transients and reduced depolarization can be fully rescued by the *Vps26-gEGFP* genomic construct. (D) Bright field sections of *Vps26^2^* and rescued *Vps26^2^* flies kept in LD for the indicated periods, or *Vps26^2^* kept in DD for 21 d. Newly eclosed *Vps26^2^* mutants are mostly normal. The UAS-*w* RNAi is expressed in the rescued flies. The PRs of *Vps26^2^* are severely impaired after 21 d in LD but remain largely intact in DD. The *Vps26-gEGFP* rescue construct rescues the PR degeneration of *Vps26^2^* mutants. Scale bar, 2 µm. (E) Quantification of intact rhabdomere numbers in (D). Thirty ommatidia from three animals were examined for each genotype. Student's *t* test; error bars are SEM; ** *p*<0.01.
(TIF)

Figure S2 Characterization of *Vps26* mutant alleles and the subcellular localization of fly Vps26 protein *in vivo.* (A) Mutations in the *XE52* alleles mapped to a 21 kb genomic region by deficiency and duplication mapping. The *Vps26* gene was tagged with *EGFP* at the C-terminus of *Vps26* in a 20 kb P[acman] genomic rescue construct (CH322-92A18; green) [132],[133]. (B) Lethal staging and rescue of *Vps26* mutant alleles. *Vps26* mutants die at pupal stages and fail to complement the *P{EP}Vps26^G2008^* allele. The *Vps26-gEGFP* genomic construct and ubiquitously expressed fly *Vps26* full-length cDNA rescue the lethality of both alleles. (C) Wingless signaling is impaired in *Vps26^1^* mutants. *Vps26^1^* mutant clones were generated in wing imaginal discs of the third instar larvae using *hh-GAL4* to drive *UAS-FLP*. Immunostaining of Wg and Sens were performed to measure Wingless signaling capacity. GFP marks wild-type control tissue. In *Vps26^1^* clones, Wg accumulates in the Wg-producing cells, and neighboring Sens expression is lost. (D) Immunostaining of Vps26 protein in mosaic wing discs of third instar larvae using an anti-fly Vps26 polyclonal antibody shows that it specifically recognizes Vps26. The *Vps26^1^* mutant clones are generated in the posterior wing disc using *hh-GAL4* and *UAS-FLP*. Wild-type control cells are marked with GFP. The antibody recognizes Vps26 punctae in the cytoplasm of wild-type cells, but no signal is observed in mutant cells. Scale bar, 10 µm. (E) Immunostaining of anti-Vps26 and anti-GFP in wing imaginal discs of third instar *Vps26-gEGFP* transgenic animals. Almost all the GFP punctae colocalize with the Vps26 punctae, documenting the specificity of the anti-Vps26 antibody. (F) Immunostaining of Vps26 and Rab7 in the wing discs of third instar control (iso) larvae. DAPI labels nuclei, and Rab7 marks late endosomes. Colocalization is quantified in (G). Scale bar, 2 µm. (G) Percent colocalization of Vps26 positive punctae with different subcellular markers in the wing discs of control animals. Six animals were examined for each staining. Error bars represent SEM.
(TIF)

Figure S3 Loss of Snx1 or Snx3 causes no or subtle phenotypes. (A) ERG traces of control (*FRT82B*), *Snx1^d1^*, or *Snx3^d1^* mosaic eyes at day 0, or kept in LD or DD for 10 d. *Snx1^d1^* mutants reveal no ERG defects upon LD for 10 d. The on-transient in *Snx3^d1^* mutants is partially lost (arrow). (B) Quantification of ERG amplitudes shown in (A). Ten flies were recorded for each genotype. Error bars represent SEM; ns, no significance. (C) Quantification of the on-transients of *Snx3^d1^* mutant PRs shown in (A). Ten flies were recorded for each genotype. Error bars represent SEM; ** *p*<0.01.
(TIF)

Figure S4 Loss of Wash in the eye does not lead to PR degeneration. (A) Wash protein levels are strongly reduced upon RNAi knockdown in the eyes of newly eclosed flies. *GMR-GAL4* drives expression of UAS-*w* RNAi together with UAS-*wash* RNAi or UAS-*GFP* RNAi (control). Scale bar, 5 µm. (B) ERG traces of flies expressing UAS-RNAi constructs against the *wash* gene or GFP at day 0 or 21 in LD. *GMR-GAL4* was used to express the UAS*-GFP* RNAi and UAS*-wash* RNAi in eyes. The UAS-*w* RNAi construct was co-expressed in all animals. The amplitudes, on-, and off-transients are not altered upon loss of *wash* when compared to control. (C) Quantification of ERG amplitudes shown in (B). Ten flies were recorded for each genotype. Error bars represent SEM; ns, no significance. (D) ERG traces of flies expressing UAS-RNAi constructs against the *wash* gene or GFP in PRs at day 0 or 21 in LD. *Rh1-GAL4* was used to express *GFP* or *wash* RNAi in R1–R6 cells. The ERG traces are not altered upon loss of *wash*. (E) Quantification of ERG amplitudes of the flies recorded in (D). Ten ERG traces were recorded for each genotype. ERG amplitudes are normalized to control PRs. Error bars represent SEM; ns, no significance.
(TIF)

Figure S5 Late endosomes and lysosomes are expanded in *Vps26* and *Vps35* mutant PRs. (A) TEM of a PR of an iso control fly kept in LD for 4 d shows normal morphology with intact rhabdomeres (R). Adherens junctions (AJ) mark PR boundaries. Mitochondria (M) and ER are indicated. Only few vesicular or membranous structures are observed. Scale bar, 0.5 µm. (B–C) Upon 4 d in LD, *Vps26^1^* mutant PRs exhibit an expansion of late endosomes (yellow arrowheads) and lysosomes (arrows). Scale bar, 0.5 µm. (D–E) Upon 4 d in LD, *Vps35^MH20^* mutant PRs show increased late endosomes (yellow arrowheads) and lysosomes (arrows). Scale bar, 0.5 µm.
(TIF)

Figure S6 Late endosomes and lysosomes are not expanded in *Vps26* or *Vps35* mutant PRs in the absence of light exposure. (A) TEM of PR of a control fly (iso) kept in DD for 4 d: note the normal morphology and intact rhabdomeres (R). Adherens junctions (AJ) mark PR boundaries. Mitochondria (M) and ER are indicated. Scale bar, 0.5 µm. (B–E) Upon 4 d in DD, *Vps26^1^* or *Vps35^MH20^* mutant PRs exhibit similar morphological features to controls shown in (A). Scale bar, 0.5 µm.
(TIF)

Figure S7 Increasing Triton X-100 concentration enhances Rh1 detection in the PR cell body. Newly eclosed iso flies were exposed to white light for 12 h. Treating the fixed eyes with 0.7% Triton X-100 prior to Rh1 immunostaining increases the detection of Rh1 in the cell body (top panels) when compared to eyes treated with 0.3% Triton X-100 (bottom panels).
(TIF)

Figure S8 PRs of control flies are not functionally or morphologically affected upon white light exposure or pulse-chase illumination. (A) ERG traces of control (iso) flies raised in the dark, exposed to white light for 12 h, exposed to white light for 12 h and recovered in the dark for 6 h, or exposed to the pulse-chase condition (20-min blue light, 5-min orange light, and then 4 h in the dark). No ERG defects were observed in the flies kept in these conditions. (B) Quantification of ERG amplitudes shown in (A). Ten flies were recorded per genotype. Error bars represent SEM; ns, no significance. (C) Bright field sections of the controls in the dark or indicated light conditions. No morphological defects were observed in the flies exposed to light. Scale bar, 2 µm. (D) Quantification of the numbers of intact rhabdomeres per ommatidium shown in (C). Thirty ommatidia from three animals were examined for each genotype. Error bars represent SEM; ns, no significance.
(TIF)

Figure S9 Rh1 accumulates in *Vps26* mutant PRs upon light exposure. (A) Immunostaining of Rh1 in the sectioned eye samples of dark-reared control (iso) or *Vps26^1^* mutants kept in the dark, exposed to white light (1,800 lux) for 12 h, or exposed to white light for 12 h and then recovered in the dark for 6 h. Both dark-reared control and *Vps26^1^* mutant PRs exhibit a normal Rh1 distribution in the rhabdomeres (top panels). Upon 12-h light exposure, both genotypes show increased Rh1 punctae in the PR cell body (arrowheads in the middle panels). Upon a 6-h recovery in the dark, more Rh1 punctae were observed in the cell body of *Vps26^1^* PRs when compared to controls (bottom panels). Scale bar, 4 µm. (B) Quantification of the numbers of Rh1 punctae per ommatidium shown in (A). Sixty ommatidia from three animals were examined for each genotype/light condition. Error bars represent SEM; * *p*<0.05; ns, no significance.
(TIF)

Figure S10 Rh1 levels are decreased upon loss of one copy of the *Rh1* gene. Western blots for Rh1 and TRP on control (iso), *Rh1^17^* heterozygous mutants, *Vps26^1^*, or *Vps26^1^;Rh1^17^*/+. Flies were raised in the dark to avoid lysosomal degradation of Rh1 induced by light. Loss of a copy of the *Rh1* gene reduces Rh1 protein levels. We loaded 0.4 fly heads in each lane.
(TIF)

Figure S11 *vps35* is expressed in vertebrate ipRGCs. (A) *vps26A* is expressed in the liver and retina of control mice. The left panel shows the ethidium bromide (EtBr)-stained total RNA in a denaturing gel. The right panel shows the results of Northern blot analysis of the same gel hybridized to radiolabeled probes for *vps26A*. Liver is the positive control for *vps26A* expression as described [136]. (B) Immunostaining of melanopsin and β-GAL. β-GAL is expressed in the Ganglion Cell Layer from the sectioned retina of control (top panels) or *vps35 lacZ* KI mice (bottom panels) at P30. β-GAL signals can be detected in ipRGCs of *vps35 lacZ* KI mice (arrow heads) but not in those of the control. Scale bar, 5 µm.
(TIF)

## Abbreviations

*cm*: *carmine*
ERG: electroretinogram
M*: metarhodopsin
*norpA*: *no receptor potential A*
PR: photoreceptor
Rh1: rhodopsin 1
TEM: transmission electron microscopy
TGN: *trans*-Golgi network
