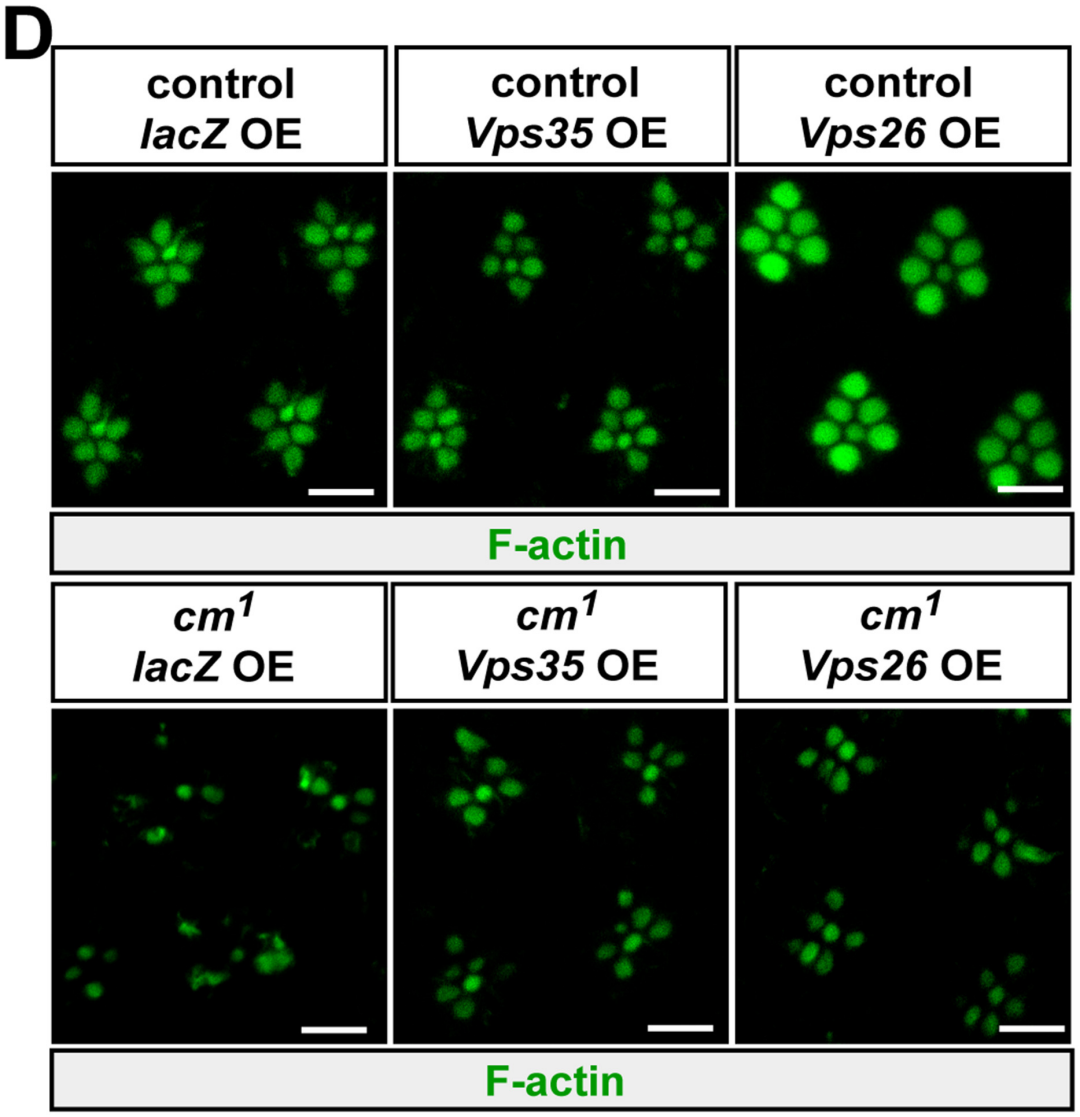# Correction: The Retromer Complex Is Required for Rhodopsin Recycling and Its Loss Leads to Photoreceptor Degeneration

**DOI:** 10.1371/journal.pbio.1002170

**Published:** 2015-05-28

**Authors:** Shiuan Wang, Kai Li Tan, Melina A. Agosto, Bo Xiong, Shinya Yamamoto, Hector Sandoval, Manish Jaiswal, Vafa Bayat, Ke Zhang, Wu-Lin Charng, Gabriela David, Lita Duraine, Kartik Venkatachalam, Theodore G. Wensel, Hugo J. Bellen

In Figure 7D, we mistakenly downloaded the wrong image for the control *Vps26* OE. This resulted in duplication of the two controls for *lacZ* OE and *Vps26* OE. The correct Figure 7D is provided here. The figure legend and the conclusion remain the same. We apologise for any inconvenience caused.

**Figure:**