# Supplementary figures and images for: The Retromer Complex Is Required for Rhodopsin Recycling and Its Loss Leads to Photoreceptor Degeneration
Source: PLoS Biol. 2014 Apr 29;12(4):e1001847. doi: 10.1371/journal.pbio.1001847 (PMC4004542; doi:10.1371/journal.pbio.1001847)

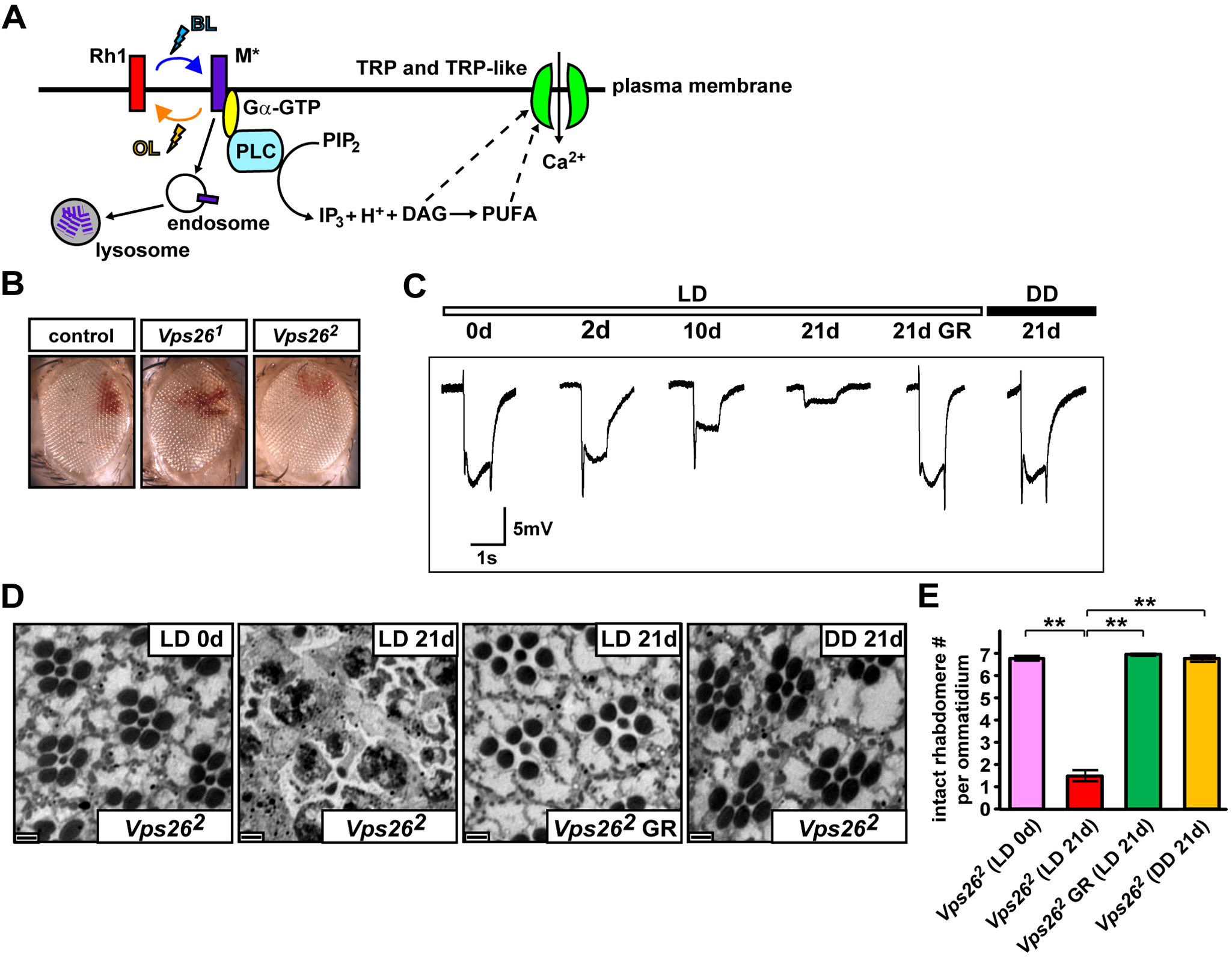

Supplement: Figure S1 — Vps262 mutants exhibit light-dependent PR degeneration. (A) Schematic of the phototransduction pathway in Drosophila. IP3, inositol 1,4,5-trisphosphate; PUFA, polyunsaturated fatty acids; BL, blue light; OL, orange light. (B) Adult mosaic eyes of control (iso), Vps261, and Vps262 mosaic mutant eyes generated by ey-FLP in a cell lethal (cl) background. White regions mark mutant tissues in the Vps26 alleles. Similar to control, Vps26 mutants exhibit large clones with normal gross eye morphology. (C) ERG traces of Vps262 mosaic eyes at day 0, kept in LD (light intensity = 1,800 lux) for 2, 10, and 21 d; Vps262 rescued by the Vps26-gEGFP genomic rescue construct kept in LD for 21 d; and Vps262 kept in DD for 21 d. The UAS-w RNAi is expressed in the rescued animals. On- and off-transients disappear and the amplitude decreases upon 2 d in LD, but remain unchanged upon keeping the flies in DD for 3 wk. The loss of on- and off-transients and reduced depolarization can be fully rescued by the Vps26-gEGFP genomic construct. (D) Bright field sections of Vps262 and rescued Vps262 flies kept in LD for the indicated periods, or Vps262 kept in DD for 21 d. Newly eclosed Vps262 mutants are mostly normal. The UAS-w RNAi is expressed in the rescued flies. The PRs of Vps262 are severely impaired after 21 d in LD but remain largely intact in DD. The Vps26-gEGFP rescue construct rescues the PR degeneration of Vps262 mutants. Scale bar, 2 µm. (E) Quantification of intact rhabdomere numbers in (D). Thirty ommatidia from three animals were examined for each genotype. Student's t test; error bars are SEM; ** p<0.01. (TIF) [file pbio.1001847.s001.tif]

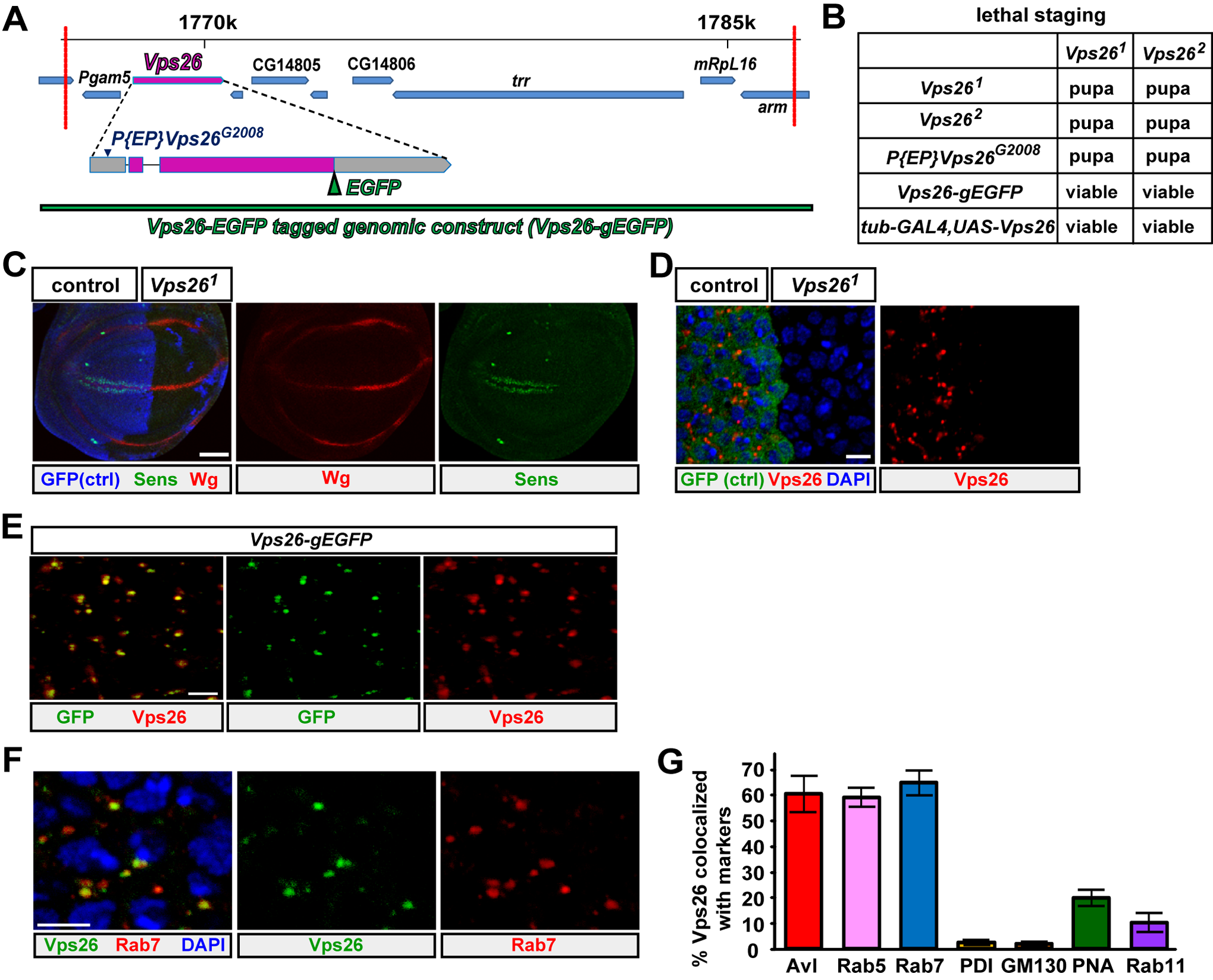

Supplement: Figure S2 — Characterization of Vps26 mutant alleles and the subcellular localization of fly Vps26 protein in vivo. (A) Mutations in the XE52 alleles mapped to a 21 kb genomic region by deficiency and duplication mapping. The Vps26 gene was tagged with EGFP at the C-terminus of Vps26 in a 20 kb P[acman] genomic rescue construct (CH322-92A18; green) [132],[133]. (B) Lethal staging and rescue of Vps26 mutant alleles. Vps26 mutants die at pupal stages and fail to complement the P{EP}Vps26G2008 allele. The Vps26-gEGFP genomic construct and ubiquitously expressed fly Vps26 full-length cDNA rescue the lethality of both alleles. (C) Wingless signaling is impaired in Vps261 mutants. Vps261 mutant clones were generated in wing imaginal discs of the third instar larvae using hh-GAL4 to drive UAS-FLP. Immunostaining of Wg and Sens were performed to measure Wingless signaling capacity. GFP marks wild-type control tissue. In Vps261 clones, Wg accumulates in the Wg-producing cells, and neighboring Sens expression is lost. (D) Immunostaining of Vps26 protein in mosaic wing discs of third instar larvae using an anti-fly Vps26 polyclonal antibody shows that it specifically recognizes Vps26. The Vps261 mutant clones are generated in the posterior wing disc using hh-GAL4 and UAS-FLP. Wild-type control cells are marked with GFP. The antibody recognizes Vps26 punctae in the cytoplasm of wild-type cells, but no signal is observed in mutant cells. Scale bar, 10 µm. (E) Immunostaining of anti-Vps26 and anti-GFP in wing imaginal discs of third instar Vps26-gEGFP transgenic animals. Almost all the GFP punctae colocalize with the Vps26 punctae, documenting the specificity of the anti-Vps26 antibody. (F) Immunostaining of Vps26 and Rab7 in the wing discs of third instar control (iso) larvae. DAPI labels nuclei, and Rab7 marks late endosomes. Colocalization is quantified in (G). Scale bar, 2 µm. (G) Percent colocalization of Vps26 positive punctae with different subcellular markers in the wing discs of con [file pbio.1001847.s002.tif]

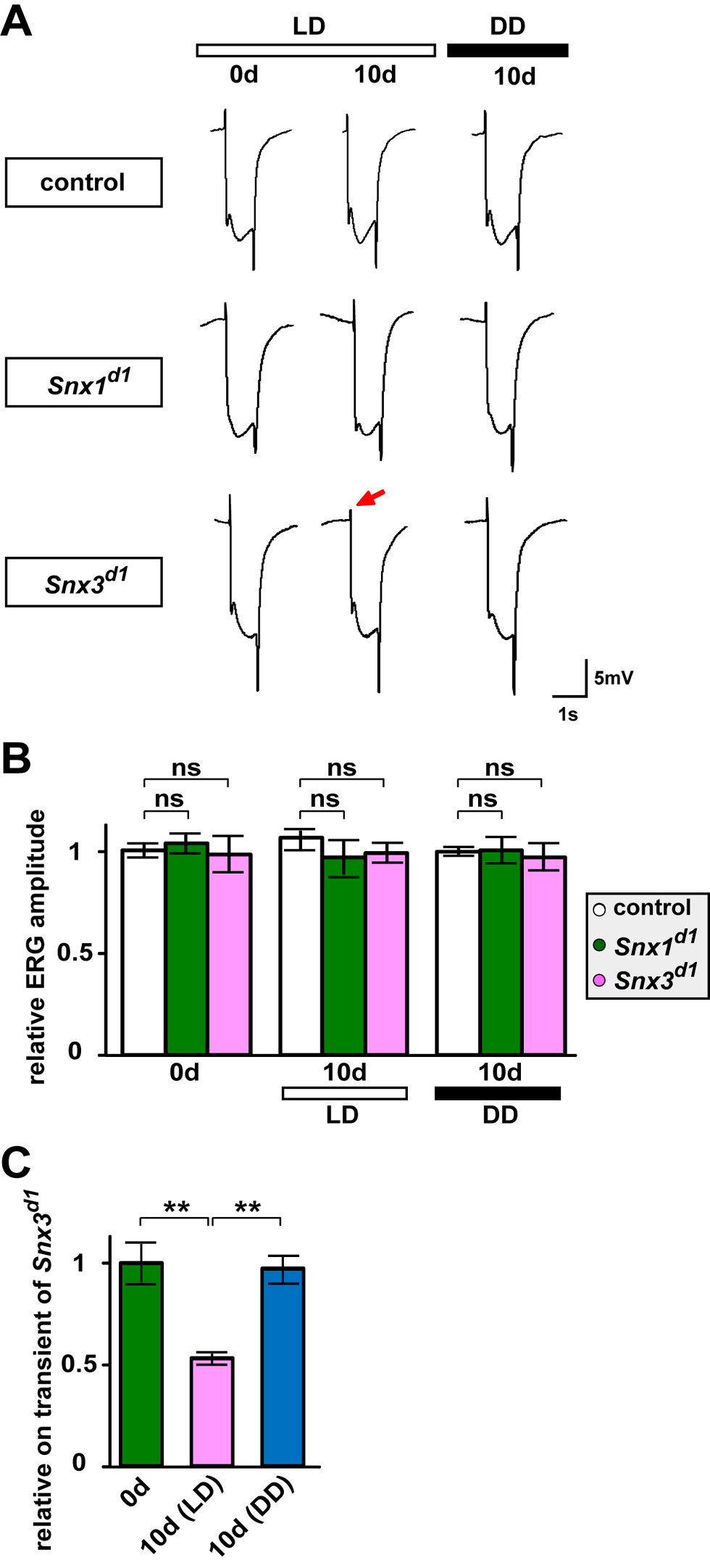

Supplement: Figure S3 — Loss of Snx1 or Snx3 causes no or subtle phenotypes. (A) ERG traces of control (FRT82B), Snx1d1, or Snx3d1 mosaic eyes at day 0, or kept in LD or DD for 10 d. Snx1d1 mutants reveal no ERG defects upon LD for 10 d. The on-transient in Snx3d1 mutants is partially lost (arrow). (B) Quantification of ERG amplitudes shown in (A). Ten flies were recorded for each genotype. Error bars represent SEM; ns, no significance. (C) Quantification of the on-transients of Snx3d1 mutant PRs shown in (A). Ten flies were recorded for each genotype. Error bars represent SEM; ** p<0.01. (TIF) [file pbio.1001847.s003.tif]

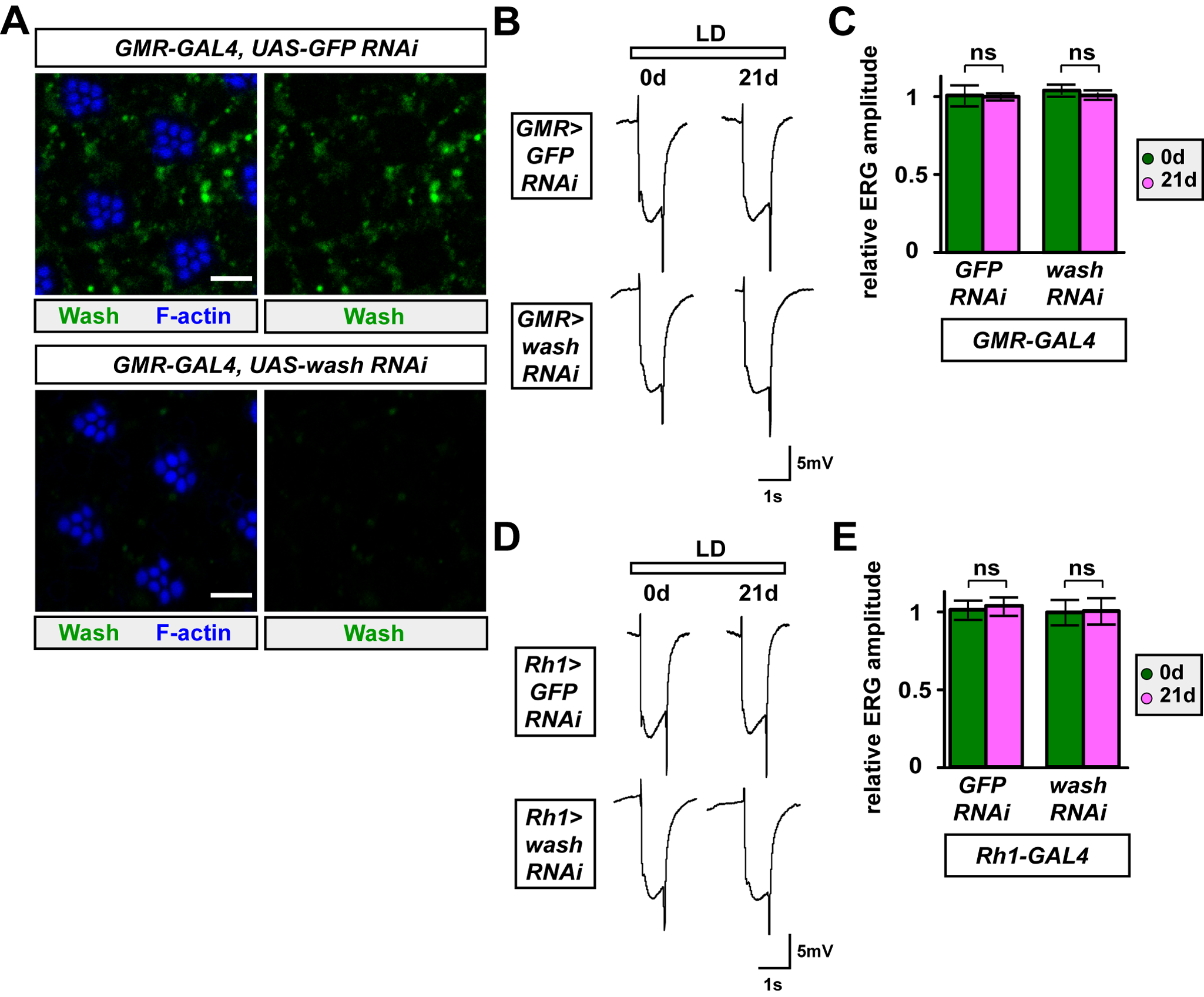

Supplement: Figure S4 — Loss of Wash in the eye does not lead to PR degeneration. (A) Wash protein levels are strongly reduced upon RNAi knockdown in the eyes of newly eclosed flies. GMR-GAL4 drives expression of UAS-w RNAi together with UAS-wash RNAi or UAS-GFP RNAi (control). Scale bar, 5 µm. (B) ERG traces of flies expressing UAS-RNAi constructs against the wash gene or GFP at day 0 or 21 in LD. GMR-GAL4 was used to express the UAS-GFP RNAi and UAS-wash RNAi in eyes. The UAS-w RNAi construct was co-expressed in all animals. The amplitudes, on-, and off-transients are not altered upon loss of wash when compared to control. (C) Quantification of ERG amplitudes shown in (B). Ten flies were recorded for each genotype. Error bars represent SEM; ns, no significance. (D) ERG traces of flies expressing UAS-RNAi constructs against the wash gene or GFP in PRs at day 0 or 21 in LD. Rh1-GAL4 was used to express GFP or wash RNAi in R1–R6 cells. The ERG traces are not altered upon loss of wash. (E) Quantification of ERG amplitudes of the flies recorded in (D). Ten ERG traces were recorded for each genotype. ERG amplitudes are normalized to control PRs. Error bars represent SEM; ns, no significance. (TIF) [file pbio.1001847.s004.tif]

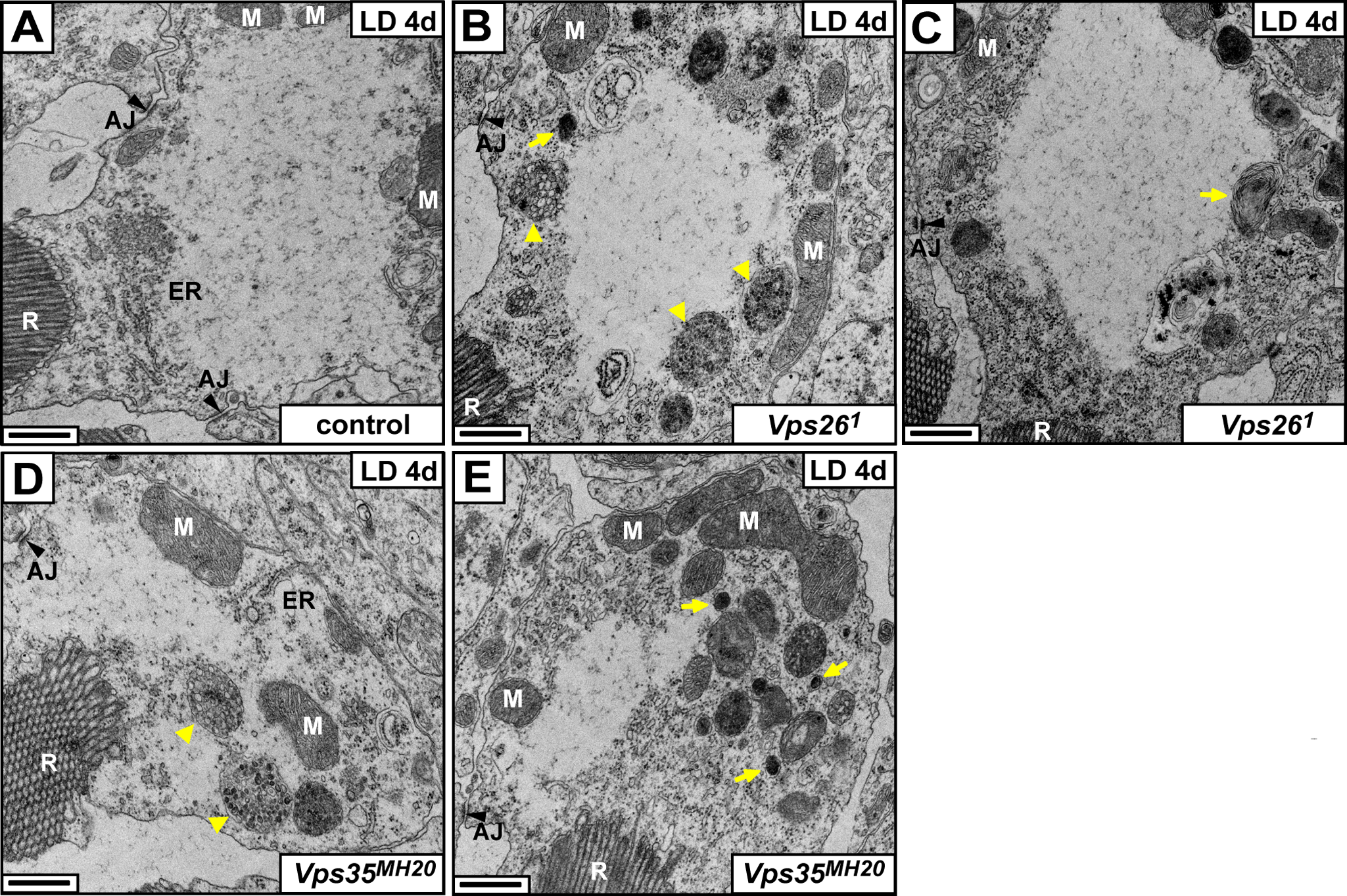

Supplement: Figure S5 — Late endosomes and lysosomes are expanded in Vps26 and Vps35 mutant PRs. (A) TEM of a PR of an iso control fly kept in LD for 4 d shows normal morphology with intact rhabdomeres (R). Adherens junctions (AJ) mark PR boundaries. Mitochondria (M) and ER are indicated. Only few vesicular or membranous structures are observed. Scale bar, 0.5 µm. (B–C) Upon 4 d in LD, Vps261 mutant PRs exhibit an expansion of late endosomes (yellow arrowheads) and lysosomes (arrows). Scale bar, 0.5 µm. (D–E) Upon 4 d in LD, Vps35MH20 mutant PRs show increased late endosomes (yellow arrowheads) and lysosomes (arrows). Scale bar, 0.5 µm. (TIF) [file pbio.1001847.s005.tif]

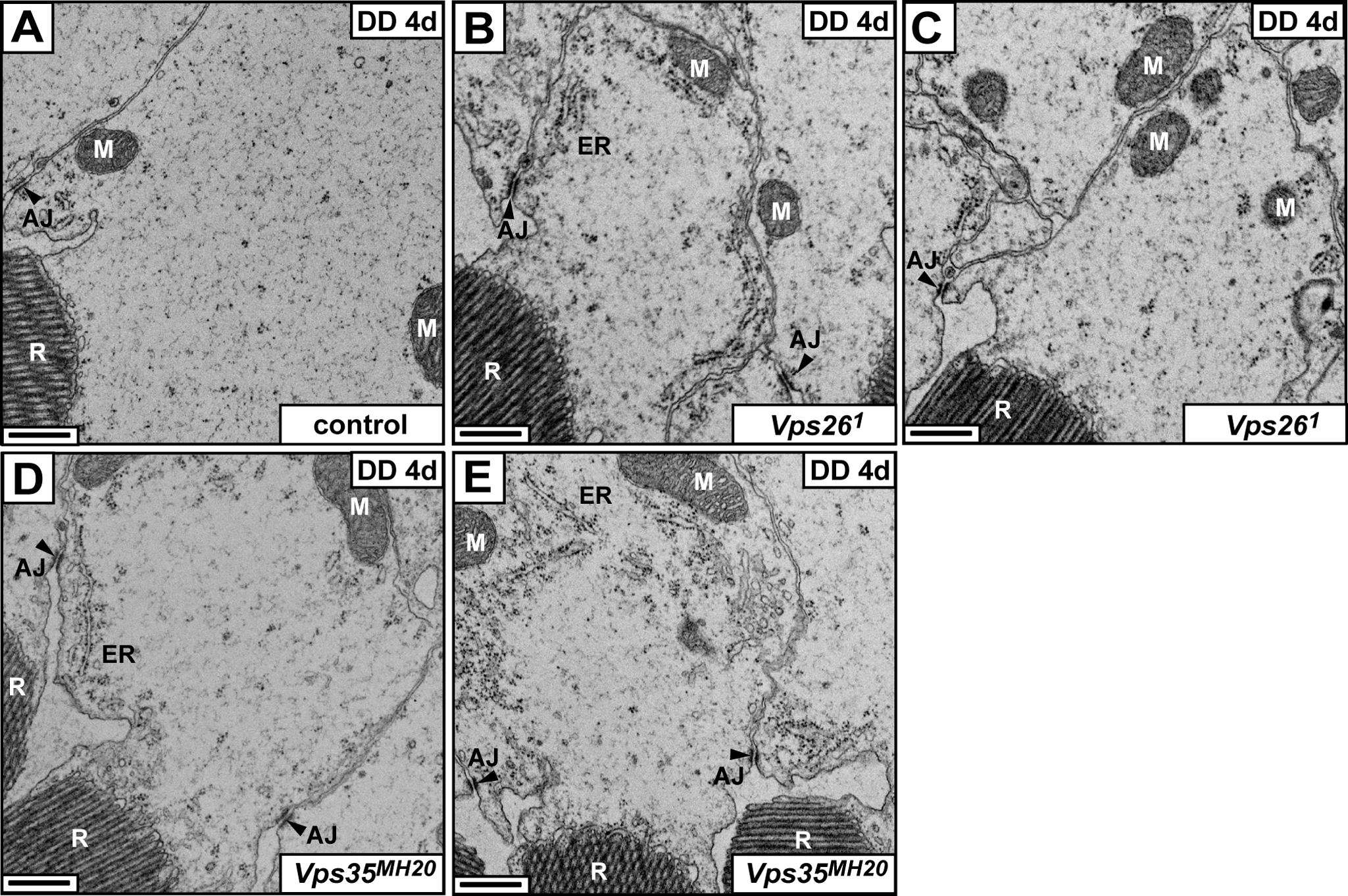

Supplement: Figure S6 — Late endosomes and lysosomes are not expanded in Vps26 or Vps35 mutant PRs in the absence of light exposure. (A) TEM of PR of a control fly (iso) kept in DD for 4 d: note the normal morphology and intact rhabdomeres (R). Adherens junctions (AJ) mark PR boundaries. Mitochondria (M) and ER are indicated. Scale bar, 0.5 µm. (B–E) Upon 4 d in DD, Vps261 or Vps35MH20 mutant PRs exhibit similar morphological features to controls shown in (A). Scale bar, 0.5 µm. (TIF) [file pbio.1001847.s006.tif]

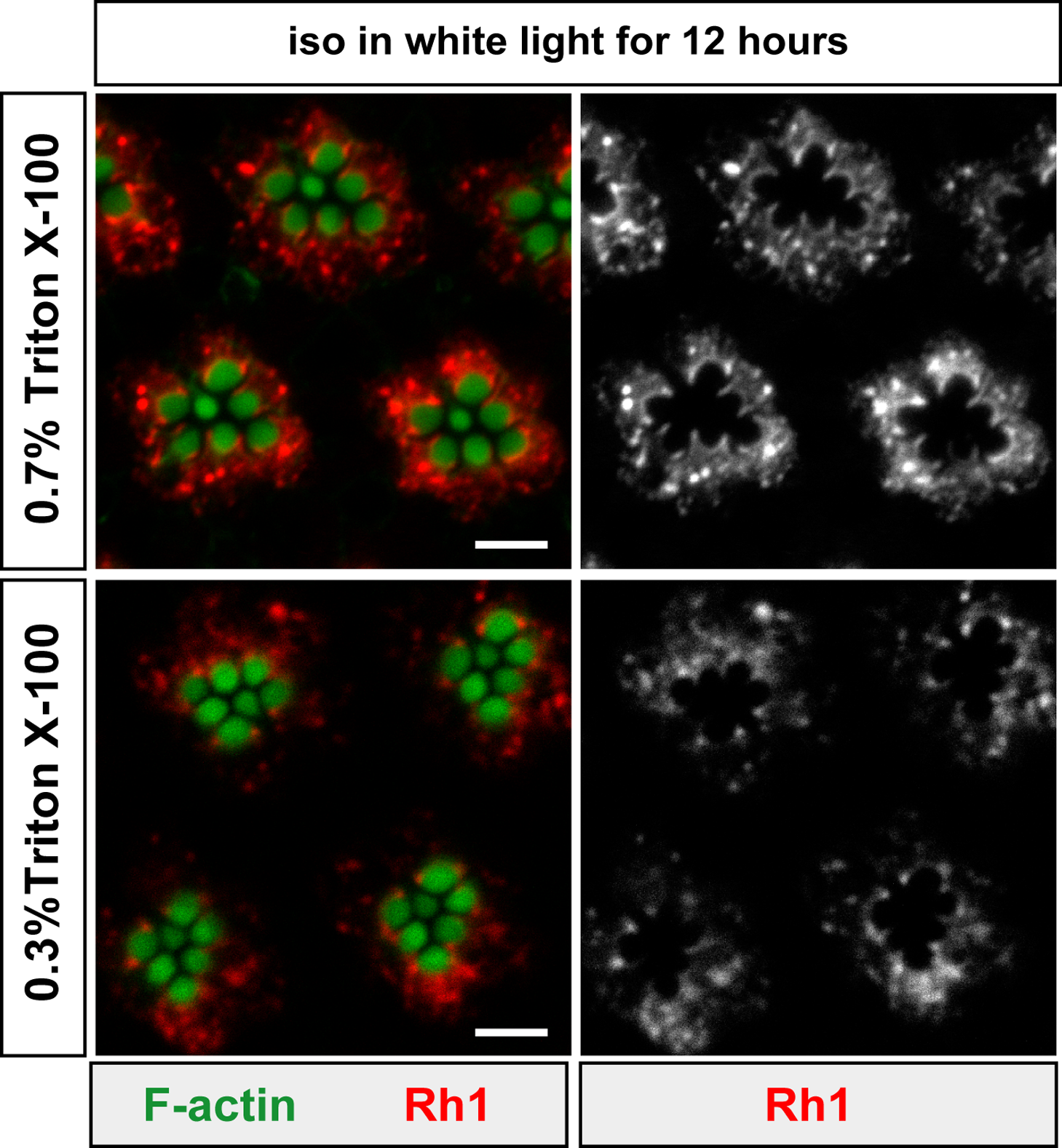

Supplement: Figure S7 — Increasing Triton X-100 concentration enhances Rh1 detection in the PR cell body. Newly eclosed iso flies were exposed to white light for 12 h. Treating the fixed eyes with 0.7% Triton X-100 prior to Rh1 immunostaining increases the detection of Rh1 in the cell body (top panels) when compared to eyes treated with 0.3% Triton X-100 (bottom panels). (TIF) [file pbio.1001847.s007.tif]

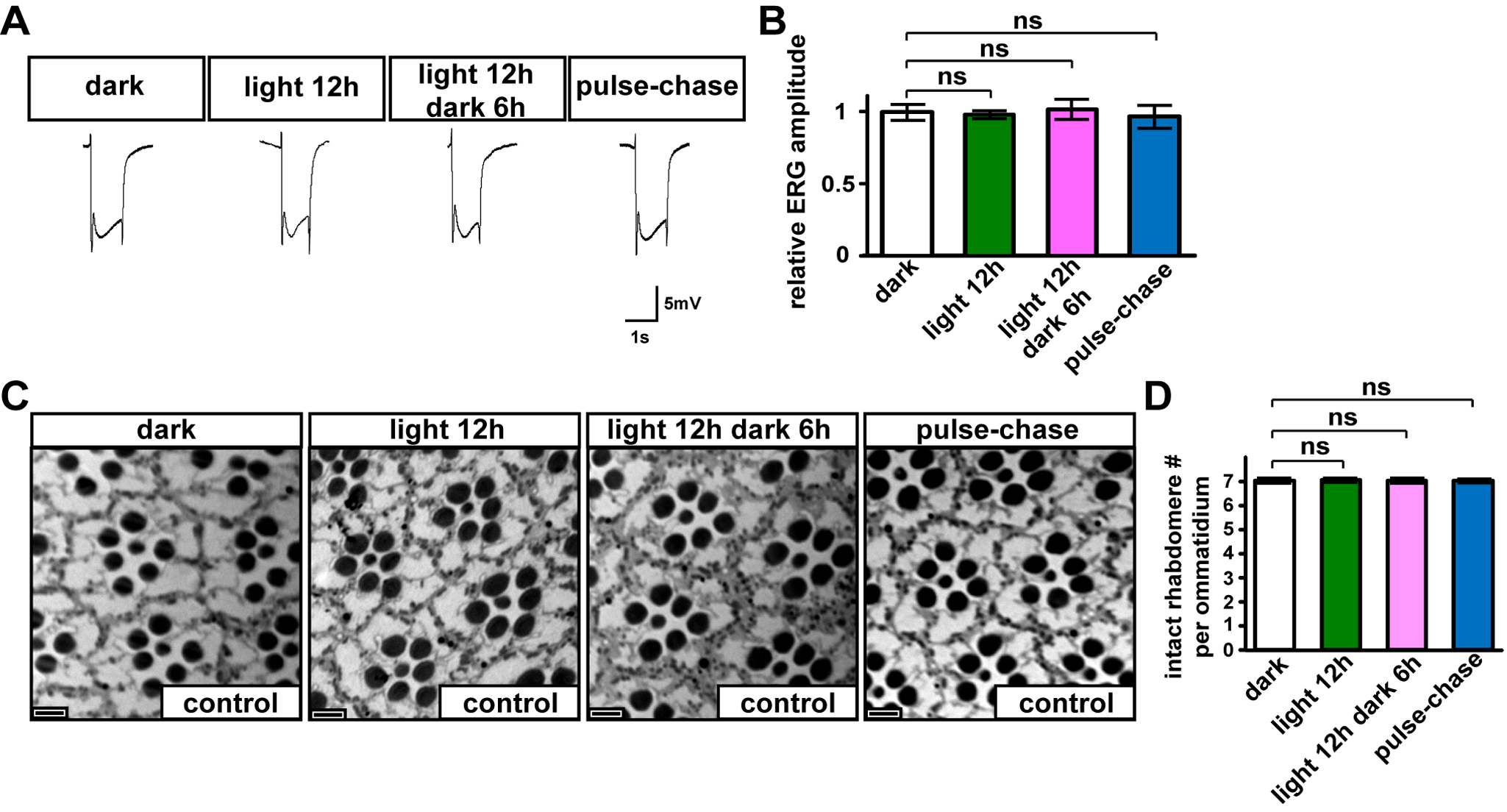

Supplement: Figure S8 — PRs of control flies are not functionally or morphologically affected upon white light exposure or pulse-chase illumination. (A) ERG traces of control (iso) flies raised in the dark, exposed to white light for 12 h, exposed to white light for 12 h and recovered in the dark for 6 h, or exposed to the pulse-chase condition (20-min blue light, 5-min orange light, and then 4 h in the dark). No ERG defects were observed in the flies kept in these conditions. (B) Quantification of ERG amplitudes shown in (A). Ten flies were recorded per genotype. Error bars represent SEM; ns, no significance. (C) Bright field sections of the controls in the dark or indicated light conditions. No morphological defects were observed in the flies exposed to light. Scale bar, 2 µm. (D) Quantification of the numbers of intact rhabdomeres per ommatidium shown in (C). Thirty ommatidia from three animals were examined for each genotype. Error bars represent SEM; ns, no significance. (TIF) [file pbio.1001847.s008.tif]

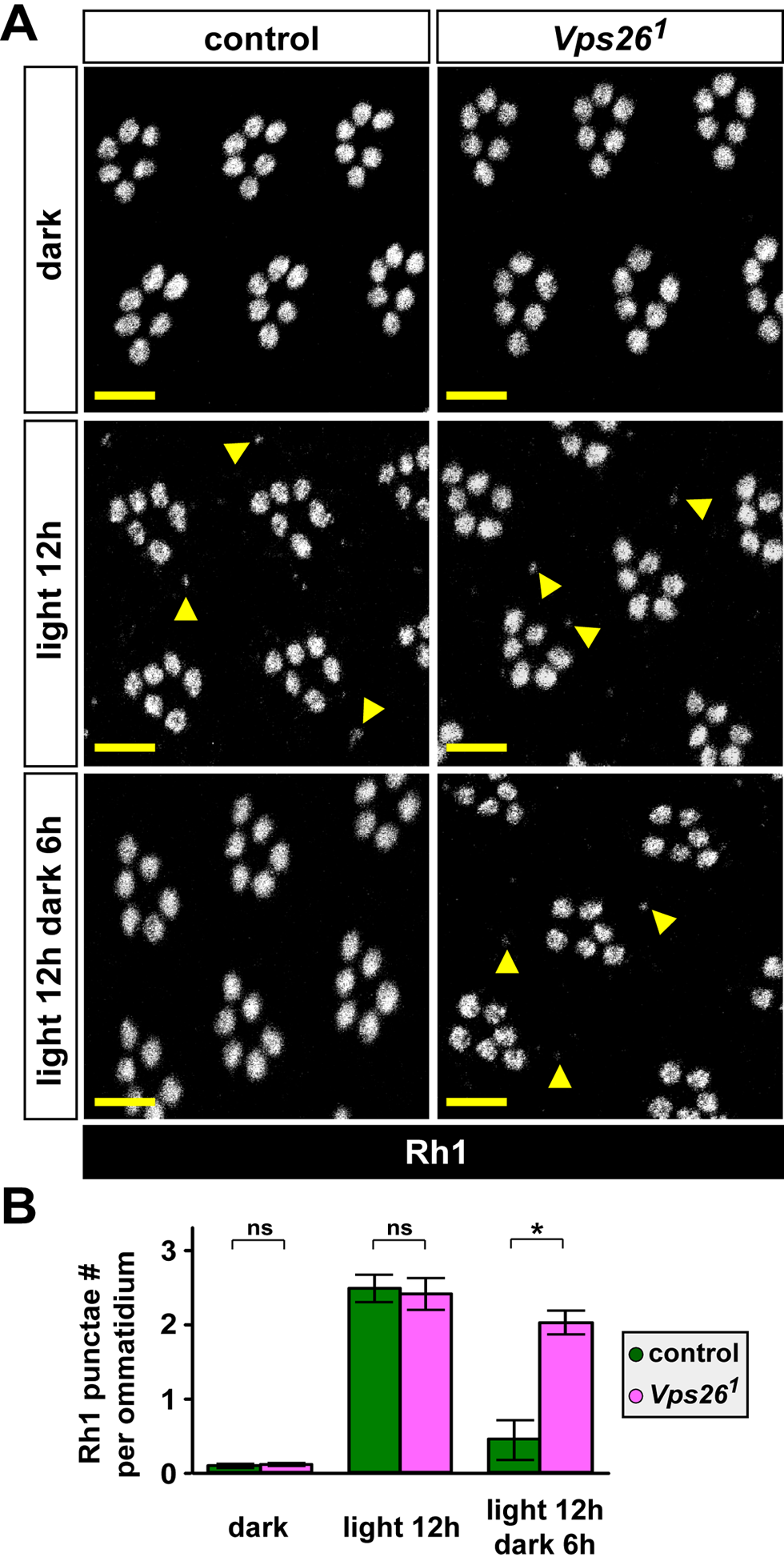

Supplement: Figure S9 — Rh1 accumulates in Vps26 mutant PRs upon light exposure. (A) Immunostaining of Rh1 in the sectioned eye samples of dark-reared control (iso) or Vps261 mutants kept in the dark, exposed to white light (1,800 lux) for 12 h, or exposed to white light for 12 h and then recovered in the dark for 6 h. Both dark-reared control and Vps261 mutant PRs exhibit a normal Rh1 distribution in the rhabdomeres (top panels). Upon 12-h light exposure, both genotypes show increased Rh1 punctae in the PR cell body (arrowheads in the middle panels). Upon a 6-h recovery in the dark, more Rh1 punctae were observed in the cell body of Vps261 PRs when compared to controls (bottom panels). Scale bar, 4 µm. (B) Quantification of the numbers of Rh1 punctae per ommatidium shown in (A). Sixty ommatidia from three animals were examined for each genotype/light condition. Error bars represent SEM; * p<0.05; ns, no significance. (TIF) [file pbio.1001847.s009.tif]

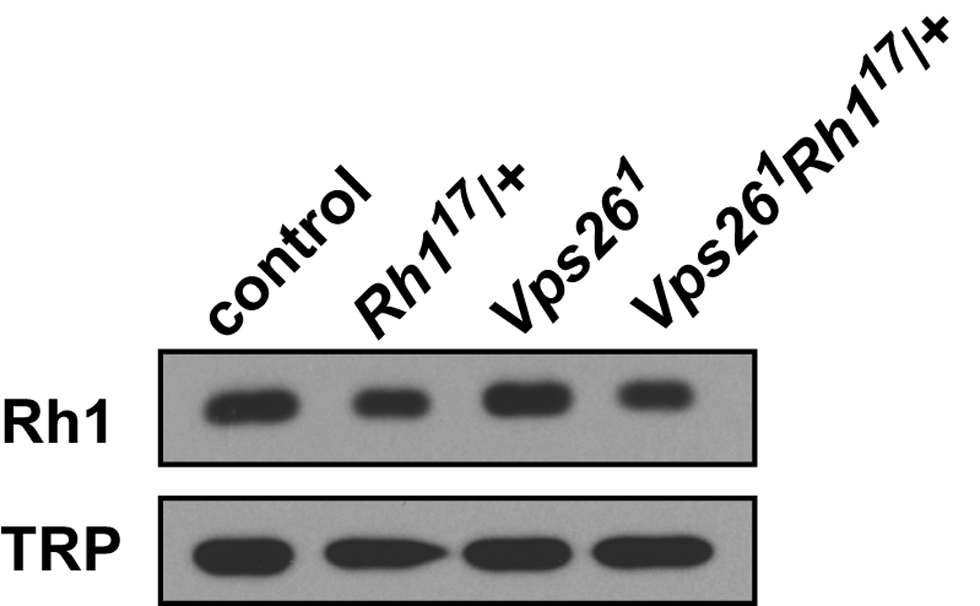

Supplement: Figure S10 — Rh1 levels are decreased upon loss of one copy of the Rh1 gene. Western blots for Rh1 and TRP on control (iso), Rh117 heterozygous mutants, Vps261, or Vps261;Rh117/+. Flies were raised in the dark to avoid lysosomal degradation of Rh1 induced by light. Loss of a copy of the Rh1 gene reduces Rh1 protein levels. We loaded 0.4 fly heads in each lane. (TIF) [file pbio.1001847.s010.tif]

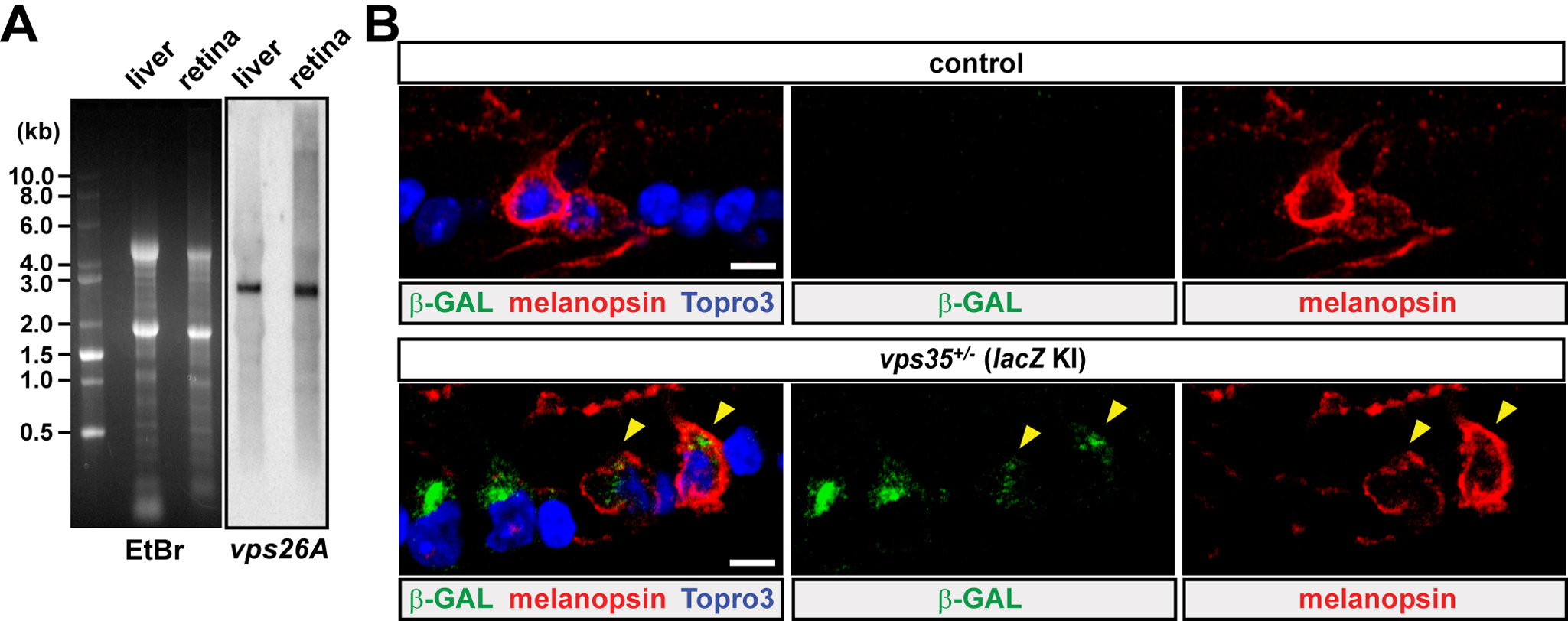

Supplement: Figure S11 — vps35 is expressed in vertebrate ipRGCs. (A) vps26A is expressed in the liver and retina of control mice. The left panel shows the ethidium bromide (EtBr)-stained total RNA in a denaturing gel. The right panel shows the results of Northern blot analysis of the same gel hybridized to radiolabeled probes for vps26A. Liver is the positive control for vps26A expression as described [136]. (B) Immunostaining of melanopsin and β-GAL. β-GAL is expressed in the Ganglion Cell Layer from the sectioned retina of control (top panels) or vps35 lacZ KI mice (bottom panels) at P30. β-GAL signals can be detected in ipRGCs of vps35 lacZ KI mice (arrow heads) but not in those of the control. Scale bar, 5 µm. (TIF) [file pbio.1001847.s011.tif]
